# Subjective Economic Inequality Decreases Emotional Intelligence, Especially for People of High Social Class

**DOI:** 10.1177/19485506211024024

**Published:** 2021-06-16

**Authors:** Anita Schmalor, Steven J. Heine

**Affiliations:** 1 8166 The University of British Columbia, Vancouver, British Columbia, Canada

**Keywords:** emotional intelligence, subjective economic inequality, subjective socioeconomic status

## Abstract

Across five studies (three preregistered; *N* = 2,481), we investigated two effects as follows: (1) Is higher subjective economic inequality associated with a decreased ability to accurately identify emotions (emotional intelligence)? When inequality is high, people are less focused on others and may thus be less motivated to correctly identify their emotions. (2) Is this main effect of subjective inequality qualified by an interaction with socioeconomic status (SES)? Past research suggests that high SES leads to lower emotional intelligence because people of higher SES are less dependent on others and thus less motivated to identify their emotions. When perceiving higher inequality, high SES individuals should feel even more self-reliant, thereby exacerbating the difference in emotional intelligence between people of low and high SES. We provide empirical support in three out of five studies for the first and in four out of five studies for the second hypothesis. An internal meta-analysis supported both hypotheses.

Humans live in social hierarchies in which their social class is based on their material wealth and status. One’s social class has far-reaching effects on health outcomes and well-being (e.g., Adler et al., 2000; Marmot, 2004), as well as in shaping basic social processes, such as how people construe others. Much past research has investigated the role of social class in emotional intelligence; one line of reasoning argues for an inverse relationship between social class and emotional intelligence (i.e., correctly inferring the emotions of others). This is because people of high socioeconomic status (SES) have a greater share of the resources, rendering them less dependent on others and less motivated to attend to them (Dietze & Knowles, 2016). That is, the greater self-sufficiency of high SES individuals makes them more self-focused (Dietze & Knowles, 2016; Kraus et al., 2010). Research supporting this finds that higher SES individuals tend to be less engaged with others (Kraus & Keltner, 2009), pay less attention to contextual cues when judging people’s emotions (Kraus et al., 2009), and are less accurate at perceiving others’ emotions (Krause et al., 2010). On the other hand, other research finds that high SES individuals are better at judging emotions (Deveney et al., 2018) or finds no association between SES and emotional intelligence (Hall et al., 2015).

These mixed findings raise the possibility that moderators may influence the relationship between SES and emotional intelligence. One such potential moderator is the degree of economic inequality that people perceive. SES and inequality are interrelated constructs that reside at different levels of description. SES is an individual-level factor that describes a person’s relative position within a hierarchy, whereas economic inequality is a macro-level factor that describes the dispersion of resources across the hierarchy. Thus, each person has their own level of SES, whereas each society has its own level of inequality. SES and inequality are conceptually interdependent as differences in SES cannot exist without some inequality, and the existence of inequality presupposes different levels of SES.

Although SES and inequality are conceptually interrelated, they are distinct constructs that may have diverging effects. Although much research has explored the links between SES and emotional intelligence (e.g., Dietze & Knowles, 2016, 2020; Kraus et al., 2010), the role of inequality remains unclear.

Why might inequality impact emotional intelligence? Here we propose and test two different effects: First, we expect that higher levels of perceived inequality will lead to lower emotional intelligence overall. In highly unequal settings, one’s position within the social hierarchy is even more consequential because people at the top receive an even larger portion of benefits. High inequality thus forms an ecology that should foster a more competitive mindset where people are motivated to reach the top because of the greater stakes and, as a result, are more self-focused and concerned about their own success. Consistent with this hypothesis, greater inequality is associated with more competition (Krupp & Cook, 2018), risk-taking (Payne et al., 2017), and independent self-concepts (Sánchez-Rodríguez et al., 2017). These correlates of inequality suggest a greater self-focus that may be associated with lower emotional intelligence. Therefore, we hypothesize that people who perceive more inequality will show less emotional intelligence overall.

Second, we expect this main effect will be qualified by an interaction between SES and perceived inequality that exacerbates the decrease in emotional intelligence for individuals of high SES. Previous research that has linked high SES with a decrease in emotional intelligence suggests that individuals of high SES are more self-sufficient and therefore need not spend as much social-cognitive resources on correctly judging the emotions of others (e.g., Dietze & Knowles, 2016, 2020; Kraus et al., 2010; but see Hall et al., 2015). As high SES individuals perceive greater inequality this effect should grow even stronger, as they should perceive an even larger gulf between themselves and the rest of the SES hierarchy, thereby leading them to view themselves as *even more* self-sufficient and, as a result, displaying *even less* emotional intelligence. In contrast, for those low in SES, perceiving more inequality does not lead them to feel any more self-sufficient compared with those who perceive little inequality. Regardless of levels of perceived inequality, people of low SES find themselves at the bottom of a hierarchy, somewhat dependent upon others and thus needing to attend more closely to them.

While each can be measured objectively, SES and inequality can also be subjectively perceived, and these feelings can influence one’s thoughts and behaviors (e.g., Adler et al., 2000; Schmalor & Heine, in press). When individuals feel that they have high rank, or that there are large differences in rank within their societies, this should affect their motivation to attend to others.

With respect to emotional intelligence, we utilize the model put forth by Mayer et al. (2016). Specifically, we focus on two aspects of the broader, four-factor model of emotional intelligence: emotional understanding (the ability to infer the emotions of others from context which we look at in Studies 1a and 1b) and emotional perception (the ability to correctly perceive the (emotions of others which we look at in Studies 2a, 2b, and 3).

In the present research, across five studies (three preregistered), we aimed to address some of the mixed findings by assessing whether high SES predicts decreased emotional intelligence and test our following two novel hypotheses: specifically, we predicted that (1) people who perceive more inequality would show less emotional intelligence and (2) that the relative worse performance of people of higher SES would be most pronounced among people who perceive high inequality. All materials, data, and analysis code are available at https://osf.io/6vseh.

## Study 1a

In Study 1a, we investigated whether SES and emotional intelligence are negatively associated, and we tested our two hypotheses.

## Method

### Sample Size

The target sample size for all five studies we conducted was based on power analysis for the expected main effect of subjective inequality on emotional intelligence. For Studies 1a and 2b, which were all correlational designs, we chose the target sample size based on recommendations from Schönbrodt and Perugini (2013; power analysis for Study 3 will be discussed in the Method section of Study 3). Simulations described by Schönbrodt and Perugini (2013) demonstrated that a sample size of 252 will allow a true correlation of ρ = .10 to be detected 80% of the time, and in all four correlational studies, we ensured that a final sample size was larger than this minimum. Because we did not consider the predicted interaction in the a priori power analyses, we report an effect size sensitivity analysis for each study in the supplemental online material (SOM).

### Participants

We solicited a convenience sample of Americans on TurkPrime and collected data from 469 participants. After excluding participants who failed attention checks our final sample consisted of 379 participants (*M*_age_ = 34.43, *SD* = 10.06; 43% female; 64% Caucasian, 22% African American, and 14% other).

## Measures

### Social Class

We operationalized SES in two ways. First, participants indicated their subjective SES (Adler et al., 2000) on a ladder with 10 rungs that indicated one’s relative standing in society (*M* = 5.53, *SD* = 1.98). Second, participants indicated which of five social classes they thought they belonged to (i.e., poor, working class, middle class, upper middle class, and upper class; Jackman & Jackman, 1983; *M* = 2.78, *SD* = .80).

### Subjective Economic Inequality and Unfairness Beliefs About Inequality

Participants completed the eight-item Subjective Inequality Scale (Schmalor & Heine, in press) which consists of two subscales: one assesses how much inequality people perceive in their state of residence (*M* = 4.57, *SD* = 1.52, Cronbach’s α = .92; e.g., “Almost all of the money that is earned goes to only a few people”) and the other assesses how unfair they find high levels of inequality to be (*M* = 5.11, *SD* = 1.33, α = .85; e.g., “It is extremely unfair if the overall amount of economic inequality is very high”) on a 7-point Likert scale from *strongly disagree* to *strongly agree* (see Table S1 in the SOM for full scale). The Subjective Inequality subscale was used to assess the association between subjective inequality and emotional intelligence, whereas the Unfairness subscale was used as a covariate.

### Emotional Intelligence

Participants took the situational test of emotional understanding (MacCann & Roberts, 2008), in which they read 42 different scenarios, and chose the emotion a target person is most likely to experience (e.g., “If the current situation continues, Denise’s employer will probably be able to move her job to a location much closer to her home, which she really wants. Denise is most likely to feel?”). We summed the number of correct responses (*M* = 22.28, *SD* = 9.00, α = .90).

### Conservatism

We measured participants’ political orientation on social issues on a 7-point scale from *very liberal* to *very conservative* (*M* = 3.66, *SD* = 1.92).

## Results and Discussion

First, we aimed to replicate the negative association between subjective SES and emotional intelligence. People who reported higher SES showed lower emotional intelligence, β *= −*4.61, *p <* .001, 95% confidence interval (–) = [−5.39, −3.83] (predictor variables in all studies are standardized; see Table S2 in the SOM for correlations of all measures).

**Figure 1.**
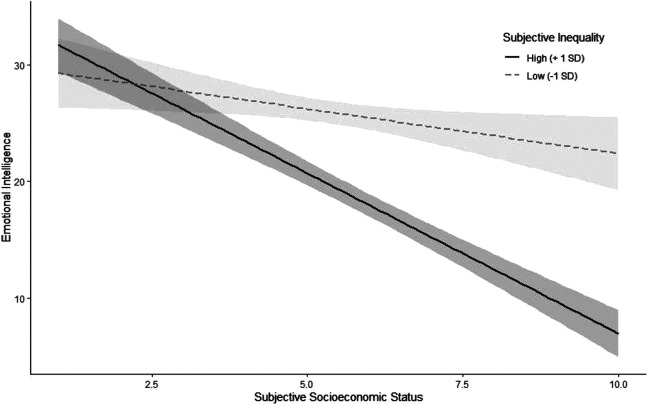
Association between SES and emotional intelligence for different levels of subjective inequality in Study 1a. Intervals around regression lines are 95% confidence intervals.

To test our first hypothesis, we predicted emotional intelligence from subjective inequality. As hypothesized, people who perceived more inequality showed worse emotional intelligence, β *= −*3.89, *p <* .001, 95% CI = [−4.71, −3.07]. To test our second hypothesis, we added an interaction term between subjective inequality and SES to the model, β *=* −1.96, *p <* .001, 95% CI = [−2.73, −1.18]. Since the interaction was significant, we probed for the simple slopes at 1 *SD* above and below the mean on subjective inequality (Figure 1). As hypothesized, this relationship was stronger for higher levels of subjective inequality; at 1 *SD* above the mean, the association between SES and emotional intelligence was β *=* −5.43, *p <* .001, whereas at 1 *SD* below the mean, it was β *=* −1.52, *p =* .019 (see Figure S1 in the SOM for the interaction decomposed by SES rather than inequality). As a robustness check, we also conducted these analyses with conservatism, gender, and unfairness beliefs about inequality as covariates because all three variables tend to be associated with subjective inequality (Schmalor & Heine, in press) and were also associated with emotional intelligence (see Table S1 in the SOM). The results hold with these covariates (see Table S3 in the SOM). To further test the robustness of these effects, we conducted the same analyses with the alternative 5-point measure of SES; the same patterns as described above emerged (see Table S4 in the SOM). Additionally, we provide the results when using objective indices of social class (viz. income, degree, and years of postsecondary education in the SOM in Tables S5–S7), where the effects are somewhat similar to those of subjective SES.

## Study 1b

In Study 1b, we sought to replicate the findings of Study 1a. We preregistered our hypotheses, methods, sample size, exclusion criteria, and the analysis plan on the OSF (https://osf.io/34zw7).

## Method

### Participants

We preregistered to collect data from 550 American participants on TurkPrime. After excluding participants who failed the attention checks the final sample consisted of 440 participants (*M*_age_ = 36.04, *SD* = 10.96; 41% female; 63% Caucasian, 20% African American, and 17% other). We did not deviate from our preregistered analyses.

## Measures

We used the same measures of social class (*M* = 5.99, *SD* = 10.96 for the ladder; *M* = 2.99, *SD* = .73 for the 5-point scale), subjective economic inequality (*M* = 4.53, *SD* = 1.46 for subjective inequality and *M* = 5.10, *SD* = 1.23 for unfairness beliefs), emotional intelligence (*M* = 21.82, *SD* = 8.96, α = .90), and conservatism (*M* = 3.74, *SD* = 1.99) as in Study 1a.

## Results and Discussion

**Figure 2.**
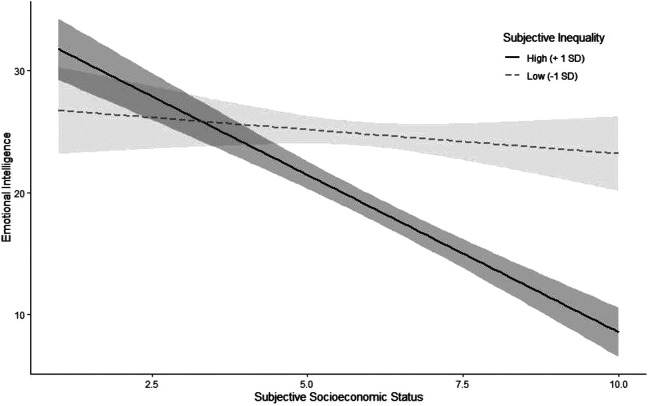
Association between socioeconomic status and emotional intelligence for different levels of subjective inequality in Study 1b. Intervals around regression lines are 95% confidence intervals.

Replicating Study 1a, we again found a negative association between SES and emotional intelligence, β *= −*3.74, *p <* .001, 95% CI = [*−*4.51, *−*2.98] (see Table S8 in the SOM for correlations of all measures). To test our first hypothesis, we predicted emotional intelligence from subjective inequality. People who perceived more inequality showed lower emotional intelligence, β *= −*2.99, *p <* .001, 95% CI = [*−*3.78, *−*2.20]. To test our second hypothesis, we added an interaction term between subjective inequality and SES to the model, β *= −*1.91, *p <* .001, 95% CI = [*−*2.63, *−*1.19]. Since the interaction was significant, we probed the interaction for the simple slopes at 1 *SD* above and below the mean on subjective inequality (Figure 2). This relationship was stronger for higher levels of subjective inequality; at 1 *SD* above the mean, the association between SES and emotional intelligence was β *= −*4.56, *p <* .001, whereas at 1 *SD* below the mean, the relationship between SES and emotional intelligence was nonsignificant, β *= −*.74, *p =* .259 (see Figure S2 in the SOM for the interaction decomposed by SES rather than inequality). As a robustness check, we also preregistered to conduct these analyses with conservatism, gender, and unfairness beliefs of high inequality as covariates. The results hold with these covariates (see Table S9 in the SOM). To further test the robustness of these effects, we also conducted the same analyses with the 5-point measure of SES and again found the same patterns (see Table S10 in the SOM). Additionally, we provide the results when using objective indices of social class (viz. income, degree, and years of postsecondary education in the SOM in Tables S11–S13), which showed a somewhat inconsistent pattern of replication.

## Study 2a

In Study 2a, we sought to replicate the results from Studies 1a and 1b using a different operationalization of emotional intelligence.

## Participants

We collected data from 284 Americans through TurkPrime (*M*_age_ = 37.34, *SD* = 13.23; 47% female; 75% Caucasian, 11% African American, and 14% other).

## Measures

### Social Class

Participants indicated their subjective SES on the same 10-rung ladder as in Study 1a (*M* = 5.34, *SD* = 2.16).

### Subjective Economic Inequality and Unfairness Beliefs About Inequality

We used the same measure of subjective inequality (*M* = 4.44, *SD* = 1.50, α = .91) and unfairness beliefs about inequality (*M* = 4.85, 1.49, α = .86) as in Study 1a.

### Emotional Intelligence

To assess emotional intelligence, participants took the “Mind-in-the-Eyes” task (Baron-Cohen et al., 2001). In this task, participants viewed 36 pictures showing only the eyes of people displaying different emotions and chose among four options which emotion the person is displaying. We summed the number of correct responses (*M* = 21.35, *SD* = 8.58, α = .91).

### Conservatism

We measured participants’ political orientation on social issues on a 7-point scale from *very liberal* to *very conservative* (*M* = 3.68, *SD* = 1.96).

We also included a number of additional measures that are part of a different project and will not be discussed here.

## Results and Discussion

**Figure 3.**
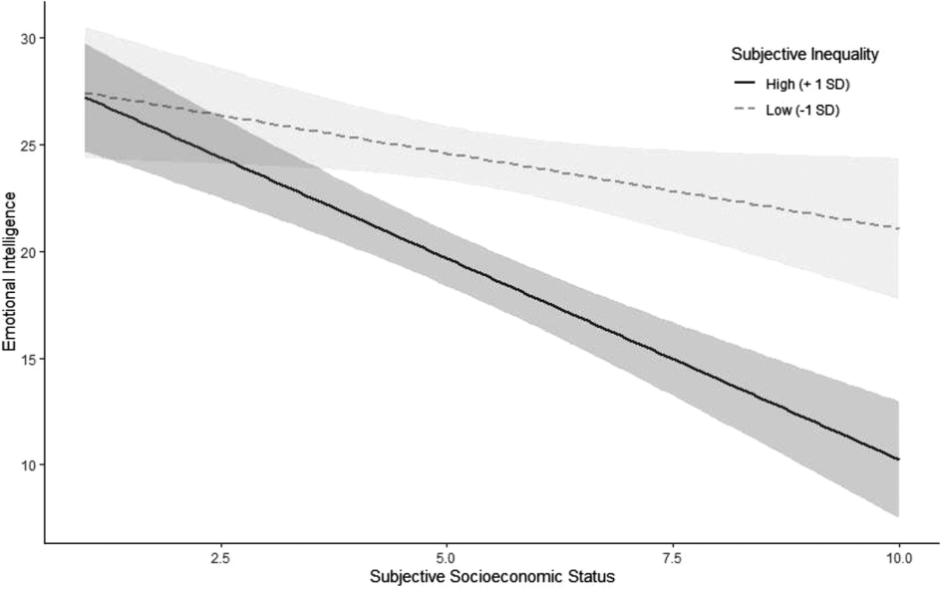
Association between socioeconomic status and emotional intelligence for different levels of subjective inequality in Study 2a. Intervals around regression lines are 95% confidence intervals.

First, we again replicated the negative association between SES and emotional intelligence, β *= −*3.11, *p <* .001, 95%CI = [*−*4.08, *−*2.13] (see Table S14 in the SOM for correlations of all measures). To test our first hypothesis, we predicted emotional intelligence from subjective inequality. As hypothesized, people who perceived more inequality had lower emotional intelligence, β *= −*2.79, *p <* .001, 95% CI = [*−*3.74, *−*1.84]. To test our second hypothesis, we added an interaction term between subjective inequality and SES to the model, β *= −*1.26, *p =* .004, 95% CI = [*−*2.101, *−*0.42]. Since the interaction was significant, we probed for the simple slopes at 1 *SD* above and below the mean on subjective inequality (Figure 3). As hypothesized, the negative association between SES and emotional intelligence was stronger at 1 *SD* above the mean of subjective inequality, β *= −*4.09, *p <* .001 than at 1 *SD* below the mean, β *= −*1.53, *p =* .035 (see Figure S3 in the SOM for the interaction decomposed by SES rather than inequality). As a robustness check, we also conducted these analyses with conservatism, gender, and unfairness beliefs about inequality as covariates. The results hold with these covariates (see Table S15 in the SOM). We further provide the results when using objective indices of social class (viz. income, degree, and years of postsecondary education in the SOM in Tables S16–S18), which showed an inconsistent pattern of replication.

## Study 2b

In Study 2b, we sought to replicate Study 2a in a community sample. We preregistered our hypotheses, methods, sample size, exclusion criteria, and the analysis plan on the OSF (https://osf.io/6yvue).

## Method

### Participants

We preregistered to collect data from a community sample in Vancouver, Canada, until reaching a final sample of 400 participants after excluding those who failed attention checks. We collected data from 564 participants and excluded 158 participants for failing attention checks or not completing the study, leaving a final sample of 406 (*M*_age_ = 35.44, *SD* = 15.90, 54% female; 58% Caucasian, 22% Asian, and 20% other). We recruited participants in different public spaces around Vancouver such as train stations, malls, and libraries. Research assistants approached people, and those who agreed to participate completed the survey on an iPad. We did not deviate from our preregistered analyses.

## Measures

We used the same measures of social class (*M* = 6.44, *SD* = 1.53), subjective economic inequality (subjective inequality *M* = 3.88, *SD* = 1.31 and unfairness beliefs *M* = 5.11, *SD* = 1.29), emotional intelligence (*M* = 25.73, *SD* = 4.47, α = .66), and conservatism (*M* = 2.79, *SD* = 1.40) as in Study 2a.

## Results and Discussion

First, we again aimed to replicate the negative association between SES and emotional intelligence; however, the two measures were not significantly related, β *=* .19, *p =* .395, 95% CI = [*−*0.25, 0.62] (see Table S19 in the SOM for correlations of all measures). Next, we tested our two main hypotheses. The analyses were preregistered both with and without political orientation, gender, SES, and subjective unfairness as covariates. Without covariates in the model, people who perceived more inequality did not show significantly lower emotional intelligence, β *= −*.12, *p =* .586, 95% CI = [*−*0.56, 0,.31]. However, when political orientation, gender, SES, and unfairness beliefs were added as covariates, greater subjective inequality was associated with lower emotional intelligence, β *= −*.60, *p =* .014, 95%CI = [*−*1.08, *−*.12]. To test our second hypothesis, we added an interaction term between subjective inequality and subjective SES to the model. Unlike as we had hypothesized, the interaction was nonsignificant, neither without covariates in the model, β *= −*.09, *p =* .696, 95%CI = [*−*0.53, 0.35], nor with covariates, β *= −*.04, *p =* .869, 95% CI = [*−*0.47, 0.40] (see Table S20 in the SOM). We further provide the results when using objective indices of social class (viz. income, degree, and years of postsecondary education in the SOM in Tables S21–S23) which yielded a mixed pattern of results.

We failed to replicate the association between SES and emotional intelligence in a community sample. However, supporting Hypothesis 1, people who perceived more inequality showed less emotional intelligence (when covariates were included). But there was no interaction between subjective inequality and SES in predicting emotional intelligence. This is less surprising given that there was no effect of SES on emotional intelligence. We will return to consider the nonsignificant results of this study in the General Discussion.

## Study 3

Some other researchers have demonstrated that perceptions of inequality can be successfully manipulated in the lab (e.g., Côté et al., 2015; Sánchez-Rodríguez et al., 2017), we explored whether we could affect people’s emotional intelligence by manipulating perceptions of inequality. The hypotheses, methods, sample size, exclusion criteria, and analysis plan are preregistered on the OSF (https://osf.io/p7v9t).

## Participants

We calculated in G × Power that a sample size of 800 participants would allow us to reliably detect a difference between two means of *d* = .20 (testing the main effect) at an α level of .05. We recruited 1,040 participants on TurkPrime, and after excluding participants who failed the attention checks specified in the preregistration, we had a final sample size of 972 American participants (*M*_age_ = 37.05, *SD* = 13.94, 63% female; 74% Caucasian, 9% African-American, and 17% other). We did not deviate from our preregistered analyses.

## Measures

### Social Class

We used the same 10-rung ladder to measure subjective SES as in Study 1a (*M* = 5.09, *SD* = 1.63).

### Manipulating Economic Inequality

Participants were randomly assigned to watch one of two 1.5-min animated videos we created. In the high inequality condition, the video argued that economic inequality in our society has increased over recent decades, whereas in the low inequality condition, the video argued that inequality has not increased over time because of increases in social spending over the past century. To strengthen the manipulation, following the video, participants were asked to describe how inequality in their society was relatively low or high.

### Emotional Intelligence

Participants completed the same Mind-in-the-Eyes task as in Study 2a (*M*= 25.65, *SD* = 4.91, α = .73).

### Conservatism

We measured participants’ political orientation on social issues on a 7-point scale from *very liberal* to *very conservative* (*M* = 3.43, *SD* = 1.71).

## Results and Discussion

After watching the video, participants responded to two questions that asked the extent to which the society they live in was unequal on a 9-point scale where higher score indicate more inequality (*M* = 6.38, *SD* = 1.92; see Table S24 in the SOM for correlations of all measures). Participants in the high inequality condition (*M* = 7.10, *SD* = 1.65) perceived more inequality than participants in the low inequality condition (*M* = 5.65, *SD* = 1.90), β *=* 1.45, *p <* .001, 95% CI = [1.23, 1.67], indicating that the video manipulation was successful.

**Figure 4.**
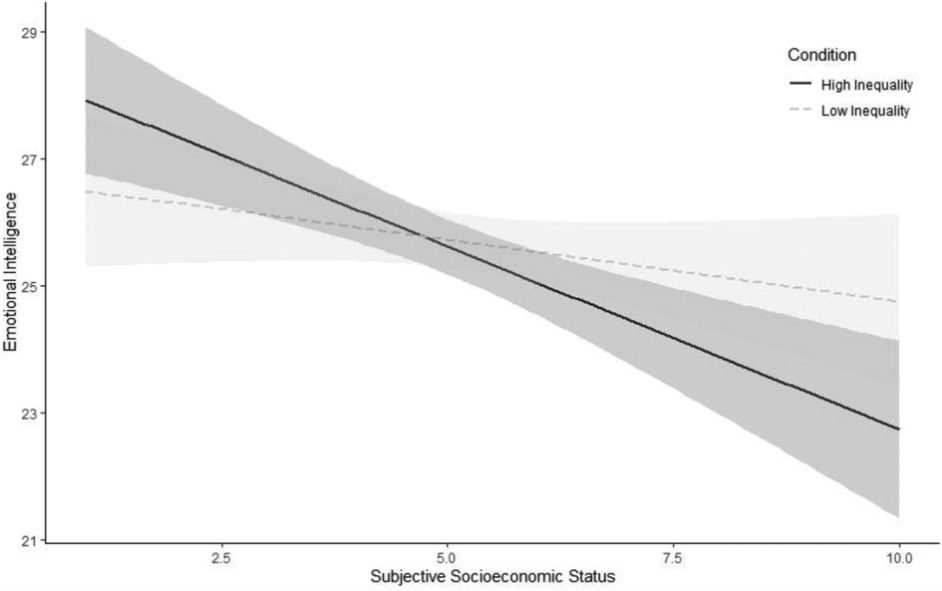
Association between socioeconomic status and emotional intelligence for low and high inequality conditions in Study 3. Intervals around regression lines are 95% confidence intervals.

Next, we tested the association between SES and emotional intelligence. We again found that people who reported higher SES showed lower emotional intelligence, β *= −*.62, *p <* .001, 95% CI = [*−*0.93, *−*0.32]. We then tested our preregistered hypotheses. In contrast to our hypothesis, people in the high inequality condition (*M* = 25.61, *SD* = 4.98) did not show significantly less emotional intelligence than participants in the low inequality condition (*M* = 25.69, *SD* = 4.85), β *= −*.08, *p =* .806, 95% CI = [*−*0.70, 0.54]. To test our second hypothesis, we added an interaction term between SES and the inequality conditions, β *= −*.62, *p =* .046, 95% CI = [*−*1.24, *−*0.01]. Since the interaction was significant, we probed for the simple slopes for the two inequality conditions (Figure 4). As hypothesized, a significant negative association between SES and emotional intelligence emerged in the high inequality condition, β *= −*.94, *p <* .001, but not in the low inequality condition, β *= −*.31, *p =* .160 (see Figure S4 in the SOM for the interaction decomposed by SES rather than inequality). As a robustness check, we also preregistered to conduct these analyses with conservatism and gender as covariates. The results hold with these covariates (although the interaction becomes only marginally significant; see Table S25 in the SOM).^1^ We further provide the results when using objective indices of social class (viz. income, degree, and years of postsecondary education in the SOM in Tables S28–S30) which did not replicate the findings.

## Internal Meta-Analysis for Studies 1–3

Across Studies 1–3, we tested whether perceptions of inequality (either measured or manipulated) were associated with emotional intelligence and whether the difference in emotional intelligence between people of low and high SES would be most pronounced at high levels of subjective inequality. The former hypothesis was supported in Studies 1a, 1b, 2a, and (partly) 2b, but not in Study 3, and the latter hypothesis was supported in Studies 1a, 1b, 2a, and 3, but not in Study 2b. To get a better estimate of the effect size for the two tests, we conducted an internal meta-analysis for each which is described in the SOM. Both a fixed-effect internal meta-analysis for the main effect of subjective inequality predicting lower emotional intelligence, *r* = *−*.20, *z = −*10.59, *p <* .001, 95% CI = [−0.23, *−*0.16], and a fixed-effect internal meta-analysis predicting the interaction between subjective inequality and SES in predicting emotional intelligence were significant, *r*_partial_ = *−*.13, *z = −*6.80, *p <* .001, 95% CI = [*−*0.17, *−*0.10] (see Figures S5 and S6 in the SOM). In addition, these meta-analyses were also found to be robust to the inclusion of covariates (see Figures S7 and S8 in the SOM) and yielded mixed results when analyzed with objective indices of social class (see Figures S9–S14 in the SOM). In conclusion, across five studies, we find evidence that greater subjective inequality is associated with less emotional intelligence and that the difference in emotional intelligence between people of low and high SES is most pronounced when subjective inequality is high.

## General Discussion

People of higher SES focus more on themselves and less on others than their lower-class counterparts and consequently have lower emotional intelligence (e.g., Dietze & Knowles, 2016; Kraus et al., 2010). Similarly, higher levels of subjective inequality make a person’s position within the rank hierarchy more consequential and shift peoples’ focus away from others and toward the self (e.g., Sánchez-Rodríguez et al., 2017). This suggests that people who perceive more economic inequality should also exhibit less emotional intelligence. In three out of five studies, people who perceived more inequality scored lower on two different measures of emotional intelligence, and these results were robust to relevant covariates. An internal meta-analysis across all studies yielded a small but significant effect.

Likewise, because perceiving greater inequality intensifies the magnitude of the differences between those who are low and high in SES, the differences in emotional intelligence among those with varying levels of SES should be more pronounced when people perceive more inequality. Indeed, in four out of five studies, the difference in emotional intelligence between people of low and high SES was exacerbated at high levels of subjective inequality. Moreover, this effect emerged across two aspects of emotional intelligence—emotional understanding (Studies 1a and 1b) and emotional perception (Studies 2a and 3), and two measures of social class which were included in Studies 1a and 1b. In two of the four studies that found a significant interaction between SES and subjective inequality, there was no significant difference in emotional intelligence for people who perceived little inequality, suggesting that the association between SES and emotional intelligence may largely disappear at low levels of subjective inequality. An internal meta-analysis across all studies yielded a small but significant effect, and these results were robust to relevant covariates. More generally, these results suggest that the effects of SES on people’s psychology may depend, in part, on the amount of inequality people perceive in their environment. When economic inequality is low, the distance between people of low and high SES is less pronounced, and therefore the psychological difference between people of low and high SES may also be less pronounced. Thus, a complete understanding of the psychological effects of SES may require considering the effects of subjective inequality. It is possible that the mixed effects of the relation between SES and emotional intelligence from past research may be a product of differences in the samples in people’s degree of subjective inequality.

While the effects we observed tended to be relatively small (internal meta-analytic *r*s = *−*.13 to *−*.20), at the societal level they may lead to substantial consequences. Indeed, many of the societal ills that have been linked to inequality (e.g., violence, depression, less trust; see Wilkinson & Pickett, 2010 for a review) are related to people’s tendencies to fail to attend to the emotions and needs of others. Perhaps one of the reasons that countries with lower inequality suffer less from these problems may be that their citizens are more attentive to the struggles of those around them.

We suggest that feelings of increased self-sufficiency and focus on the self are the potential mechanisms that may underlie the interaction between SES and perceptions of inequality; however, we have not tested them. One potential alternative explanation for the observed effects could be that people of higher SES are generally less motivated to perform well on tests—irrespective of whether the tests are specific to social cognitive performance. However, other research finds that participants of higher SES perform comparably or better on tasks not related to emotional intelligence (such as object recognition; Dietze & Knowles, 2016, 2020; or cognitive tasks; Mani et al., 2013) suggesting that this is an unlikely account.

These studies were conducted exclusively with North American samples which limits their generalizability. In particular, because four of the five studies were conducted in the United States, which has among the highest inequality among industrialized countries (Gini = .391; data from the Organisation for Economic Co-operation and Development, 2018), the results may not generalize to less unequal countries. The only study (Study 2b) that did not replicate the finding that high SES predicted lower emotional intelligence was conducted in Canada (Gini = .307; data from the Organisation for Economic Co-operation and Development, 2018). We also found that the Canadian sample in Study 2b perceived significantly less inequality (*M* = 3.88) than the American sample in Study 2a (*M* = 4.44; *b* = *−*.56, *p <* .001), and this difference may have contributed to this failed replication. In addition, the reliability for the Mind-in-the-Eyes test in Study 2b (.66) was considerably lower than in Study 2a (.91), suggesting poor data quality. Furthermore, all of the studies that found the hypothesized effects were conducted online, and future research would benefit from exploring samples collected in more diverse settings.

These findings focused only on perceptions of economic inequality. Subjective inequality and objective indices of inequality have been found to be weakly to moderately correlated (see Schmalor & Heine, in press). This is not so surprising given that objective indices of inequality are calculated based on the distribution of income/wealth in a specific geographic area while subjective inequality is informed by other aspects of people’s lives such as their SES, political orientation, and media habits (e.g., Diermeier et al., 2017). Future research would benefit from exploring how these results compare to studies looking at objective indices of inequality.

Furthermore, we focused on the subjective experience of SES, yet to get a complete understanding of the relationship between SES and emotional intelligence, it is important to also look at objective SES indices such as income and education. We presented the results with those in the SOM, and while these results tend to be consistent (although not as robust) with subjective SES, they also point to the need to further investigate when and why objective and subjective measures of social class diverge in their predictions.

## Supplemental Material

Supplemental Material, sj-docx-1-spp-10.1177_19485506211024024 - Subjective Economic Inequality Decreases Emotional Intelligence, Especially for People of High Social Class Click here for additional data file. Supplemental Material, sj-docx-1-spp-10.1177_19485506211024024 for Subjective Economic Inequality Decreases Emotional Intelligence, Especially for People of High Social Class by Anita Schmalor and Steven J. Heine in Social Psychological and Personality Science

## Note

1. We also preregistered two additional more liberal exploratory analyses in which we planned to exclude participants who were not convinced by the arguments made in the video. Although these analyses (partly) support our hypotheses, in retrospect, we believe that these exclusion criteria render the samples nonrandom. These analyses are in Tables S26 and S27 in the SOM.
