# Supplementary material for: Subjective Economic Inequality Decreases Emotional Intelligence, Especially for People of High Social Class
Source: Soc Psychol Personal Sci. 2021 Jun 16;13(2):608–17. doi: 10.1177/19485506211024024 (PMC8892066; doi:10.1177/19485506211024024)
Supplement: Supplemental Material, sj-docx-1-spp-10.1177_19485506211024024 - Subjective Economic Inequality Decreases Emotional Intelligence, Especially for People of High Social Class [file sj-docx-1-spp-10.1177_19485506211024024.docx]

**Supplementary Online Materials for**

**Subjective Economic Inequality Decreases Emotional Intelligence, Especially for People of High Social Class**

**Supplementary Online Materials for this manuscript include the following:**

Effect-size sensitivity analyses

Tables S1 to S30

Figures S1 to S6

**Effect-size sensitivity** **analyses**

**Study 1a.** To determine whether the sample size of 379 participants was sufficient to detect an interaction, we did an effect-size sensitivity analysis. For an effect-size sensitivity analysis, the achieved (or anticipated) sample size and desired power are used to calculate the minimum population effect size that can be detected with or above that power (Giner-Sorolla et al., 2021). To conduct the effect-size sensitivity analysis, we used the paramtest package (Hughes, 2017). With the paramtest package, researchers can simulate an interaction and get power estimates for the main effects and the interaction term in a model. We provided the effect sizes for the two main effects (i.e., subjective inequality and SES in the interaction model) as input. Because the power estimates are the output, we adjusted the effect size for the interaction term until the simulation calculated a power estimate of 80% for the interaction term (with 10,000 simulations). At a sample size of 379 participants and around 80% power, the minimum detectable population effect size is -0.124 which is well below the effect size observed in this study suggesting that the study was well powered to detect an interaction effect.

**Study 1b.** To determine whether the sample size was sufficient to detect an interaction, we did an effect-size sensitivity analysis following the procedure described in Study 1a. At a sample size of 440 participants and around 80% power, the minimum detectable population effect size is -0.122 which is well below the effect size observed in this study suggesting that the study was well powered to detect an interaction effect.

**Study 2a.** To determine whether the sample size was sufficient to detect an interaction, we did an effect-size sensitivity analysis following the procedure described in Study 1a. At a sample size of 253 participants and around 80% power, the minimum detectable population effect size is -0.161 which is slightly above the effect size observed in this study suggesting that the study may have been somewhat underpowered.

**Study 2b.** To determine whether the sample size was sufficient to detect an interaction, we did an effect-size sensitivity analysis following the procedure described in Study 1a. At a sample size of 406 participants and around 80% power, the minimum detectable population effect size is -0.142 which is considerably higher than the effect size observed in this study suggesting that the study may have been somewhat underpowered.

**Study 3.** To determine whether the sample size was sufficient to detect an interaction, we did an effect-size sensitivity analysis following the procedure described in Study 1a. At a sample size of 972 participants and around 80% power, the minimum detectable population effect size is -0.091 which is below the sample size observed in this study suggesting that the study was adequately powered for an interaction effect.

| **Inequality subscale** |
| --- |
| Almost all of the money that is earned goes to only a few people. |
| Besides those at the very top, no one else has much money at all. |
| Real opportunities to succeed in life are only available to the wealthy. |
| Only those at the top own any wealth at all. |
| **Unfairness subscale** |
| It is extremely unfair if the overall amount of economic inequality is very high. |
| It is not fair at all if there are large differences in income between the rich and poor. |
| It is immoral if your income is dependent on where you grew up. |
| It is extremely unjust if children of affluent parents get a better education. |

**Table S1.** Subjective Inequality Scale. Participants read the following instructions: Please indicate how much you agree or disagree with the following statements. We want to know how you feel about these statements. There are no right or wrong answers. When responding to these statements, please consider how well you think they describe the state you currently live in. All items are on a 7-point scale: (1 = Strongly disagree, 7 = Strongly agree).

|  | *Emotional Intelligence* | *Subjective Inequality* | *Subjective*  *Socioeconomic Status* | *Social Class* | *Income* | *Degree* | *Years of*  *Postsecondary Education* | *Unfairness*  *Beliefs* | *Conservatism* | *Age* | *Gender* |
| --- | --- | --- | --- | --- | --- | --- | --- | --- | --- | --- | --- |
| *Emotional*  *Intelligence* | .90 |  |  |  |  |  |  |  |  |  |  |
| *Subjective*  *Inequality* | -0.435 *(<.001)* | .92 |  |  |  |  |  |  |  |  |  |
| *Subjective*  *Socioeconomic Status* | -0.510 *(<.001)* | 0.126 *(.014)* | - |  |  |  |  |  |  |  |  |
| *Social*  *Class* | -0.394 *(<.001)* | 0.060 *(.245)* | 0.734 *(<.001)* | - |  |  |  |  |  |  |  |
| *Income* | -0.110 *(.032)* | -0.028 *(.584)* | 0.511 *(<.001)* | 0.543 *(<.001)* | - |  |  |  |  |  |  |
| *Degree* | -0.376 *(<.001)* | 0.212 *(<.001)* | 0.477 *(<.001)* | 0.421 *(<.001)* | 0.284 *(<.001)* | - |  |  |  |  |  |
| *Years of*  *Postsecondary Education* | -0.102 *(.047)* | 0.072 *(.163)* | 0.132 *(.010)* | 0.028 *(.587)* | 0.022 *(.669)* | 0.086 *(.093)* | - |  |  |  |  |
| *Unfairness*  *Beliefs* | -0.202 *(<.001)* | 0.575 *(<.001)* | 0.066 *(.202)* | 0.011 *(.827)* | -0.036 *(.487)* | 0.145 *(.005)* | 0.058 *(.263)* | .85 |  |  |  |
| *Conservatism* | -0.460 *(<.001)* | 0.024 *(.635)* | 0.234 *(<.001)* | 0.179 *(<.001)* | 0.059 *(.253)* | 0.106 *(.039)* | 0.095 *(.065)* | -0.177 *(.001)* | - |  |  |
| *Age* | 0.258 *(<.001)* | -0.243 *(<.001)* | -0.036 *(.482)* | -0.024 *(.644)* | 0.075 *(.146)* | -0.050 *(.336)* | -0.027 *(.603)* | -0.147 *(.004)* | -0.057 *(.268)* | - |  |
| *Gender* | -0.302 *(<.001)* | 0.181 *(<.001)* | 0.232 *(<.001)* | 0.158 *(.002)* | 0.032 *(.541)* | 0.185 *(<.001)* | 0.049 *(.338)* | 0.078 *(.128)* | 0.132 *(.010)* | -0.132 *(.010)* | - |
| *Computed correlation used pearson-method with listwise-deletion.* | | | | | | | | | | | |

**Table S2.** Correlation between all measures in Study 1a (with Cronbach’s alpha on diagonal) in Study 1a. Income: 1=$15,000 or less, 2=$15,001-$25,000, 3=$25,001-$35,000, 4=$35,001-$50,000, 5=$50,001-$75,000, 6=$75,001-$100,000, 7=$100,001-$150,000, 8=$150,000 or more (measured as annual household income before tax); Degree: 1=no formal education, 2=elementary school, 3=junior high school, 4=senior high school or GED, 5=2-year college degree, 6=4-year college degree, 7=master's degree, 8=doctoral degree; Years of postsecondary education: Participants were asked to indicate how many years of postsecondary education they have taken; Conservatism: 1= very liberal, 7 = very conservative; Gender coded as 0 = female, 1 = male.

**
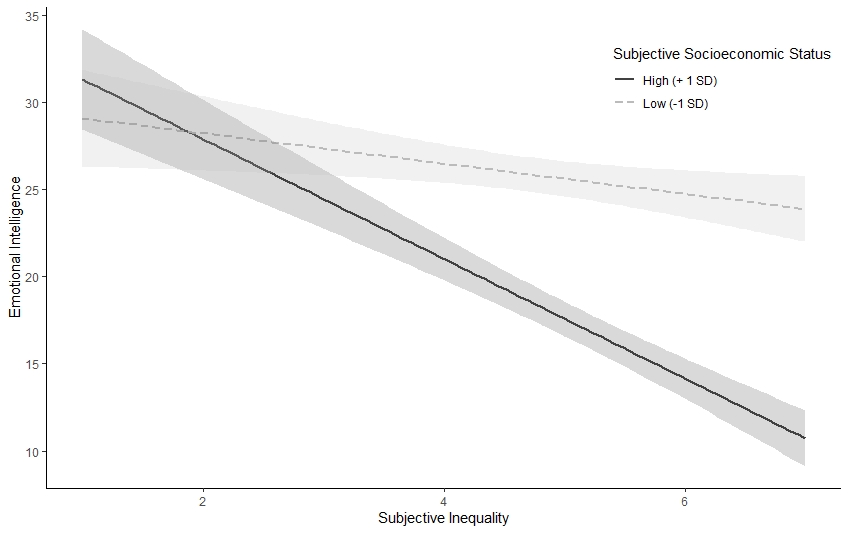
**

**Figure S1.** Association between subjective inequality and emotional intelligence at + 1SD (b=-5.24, p<.001) and at -1SD of SES (b=-1.32, p=0.016) in Study 1a. Intervals around regression lines are 95% confidence intervals.

|  | **Emotional Intelligence Model 1** | | | **Emotional Intelligence Model 2** | | | **Emotional Intelligence Model 3** | | | **Emotional Intelligence Model 4** | | | **Emotional Intelligence Model 5** | | | **Emotional Intelligence Model 6** | | |
| --- | --- | --- | --- | --- | --- | --- | --- | --- | --- | --- | --- | --- | --- | --- | --- | --- | --- | --- |
| *Predictors* | *Estimates* | *CI* | *p* | *Estimates* | *CI* | *p* | *Estimates* | *CI* | *p* | *Estimates* | *CI* | *p* | *Estimates* | *CI* | *p* | *Estimates* | *CI* | *p* |
| (Intercept) | 22.28 | 21.51 – 23.06 | **<0.001** | 24.00 | 22.91 – 25.08 | **<0.001** | 22.28 | 21.47 – 23.10 | **<0.001** | 23.41 | 22.44 – 24.38 | **<0.001** | 22.52 | 21.84 – 23.21 | **<0.001** | 23.52 | 22.57 – 24.48 | **<0.001** |
| Subjective SES | -4.61 | -5.39 – -3.83 | **<0.001** | -3.52 | -4.26 – -2.78 | **<0.001** |  |  |  | -3.20 | -3.86 – -2.54 | **<0.001** | -3.47 | -4.22 – -2.73 | **<0.001** | -2.77 | -3.46 – -2.08 | **<0.001** |
| Conservatism |  |  |  | -3.11 | -3.84 – -2.39 | **<0.001** |  |  |  | -3.24 | -3.90 – -2.58 | **<0.001** |  |  |  | -2.99 | -3.65 – -2.32 | **<0.001** |
| Gender |  |  |  | -3.01 | -4.47 – -1.55 | **<0.001** |  |  |  | -1.96 | -3.27 – -0.64 | **0.004** |  |  |  | -1.86 | -3.15 – -0.57 | **0.005** |
| Subjective Inequality |  |  |  |  |  |  | -3.89 | -4.71 – -3.07 | **<0.001** | -3.05 | -3.83 – -2.27 | **<0.001** | -3.28 | -3.97 – -2.59 | **<0.001** | -3.10 | -3.87 – -2.34 | **<0.001** |
| Unfairness Beliefs |  |  |  |  |  |  |  |  |  | -0.35 | -1.14 – 0.43 | 0.382 |  |  |  | -0.16 | -0.94 – 0.62 | 0.684 |
| Subjective SES* Subjective Inequality |  |  |  |  |  |  |  |  |  |  |  |  | -1.96 | -2.73 – -1.18 | **<0.001** | -1.33 | -2.04 – -0.61 | **<0.001** |
| Observations | 379 | | | 378 | | | 379 | | | 378 | | | 379 | | | 378 | | |
| R^2^ / adjusted R^2^ | 0.262 / 0.260 | | | 0.408 / 0.404 | | | 0.187 / 0.185 | | | 0.535 / 0.529 | | | 0.438 / 0.433 | | | 0.551 / 0.543 | | |

**Table S3.** Emotional intelligence predicted from SES on ladder scale and subjective inequality including covariates in Study 1a. Conservatism: 1= very liberal, 7 = very conservative; Gender coded as 1 = male, 0 = female; SES = socioeconomic status. Conservatism, SES, unfairness beliefs, and subjective inequality are standardized.

|  | **Emotional Intelligence Model 1** | | | **Emotional Intelligence Model 2** | | | **Emotional Intelligence Model 3** | | | **Emotional Intelligence Model 4** | | | **Emotional Intelligence Model 5** | | | **Emotional Intelligence Model 6** | | |
| --- | --- | --- | --- | --- | --- | --- | --- | --- | --- | --- | --- | --- | --- | --- | --- | --- | --- | --- |
| *Predictors* | *Estimates* | *CI* | *p* | *Estimates* | *CI* | *p* | *Estimates* | *CI* | *p* | *Estimates* | *CI* | *p* | *Estimates* | *CI* | *p* | *Estimates* | *CI* | *p* |
| (Intercept) | 22.28 | 21.45 – 23.12 | **<0.001** | 24.41 | 23.29 – 25.54 | **<0.001** | 22.28 | 21.47 – 23.10 | **<0.001** | 23.73 | 22.72 – 24.73 | **<0.001** | 22.38 | 21.65 – 23.11 | **<0.001** | 23.78 | 22.79 – 24.77 | **<0.001** |
| Social Class | -3.57 | -4.40 – -2.73 | **<0.001** | -2.64 | -3.40 – -1.89 | **<0.001** |  |  |  | -2.52 | -3.19 – -1.85 | **<0.001** | -2.93 | -3.69 – -2.17 | **<0.001** | -2.23 | -2.91 – -1.55 | **<0.001** |
| Conservatism |  |  |  | -3.41 | -4.16 – -2.67 | **<0.001** |  |  |  | -3.53 | -4.21 – -2.85 | **<0.001** |  |  |  | -3.36 | -4.04 – -2.68 | **<0.001** |
| Gender |  |  |  | -3.73 | -5.24 – -2.23 | **<0.001** |  |  |  | -2.51 | -3.86 – -1.16 | **<0.001** |  |  |  | -2.46 | -3.80 – -1.13 | **<0.001** |
| Subjective Inequality |  |  |  |  |  |  | -3.89 | -4.71 – -3.07 | **<0.001** | -3.16 | -3.97 – -2.35 | **<0.001** | -3.53 | -4.27 – -2.79 | **<0.001** | -3.16 | -3.96 – -2.36 | **<0.001** |
| Unfairness Beliefs |  |  |  |  |  |  |  |  |  | -0.50 | -1.32 – 0.32 | 0.232 |  |  |  | -0.31 | -1.12 – 0.51 | 0.460 |
| Social Class* Subjective Inequality |  |  |  |  |  |  |  |  |  |  |  |  | -1.70 | -2.51 – -0.89 | **<0.001** | -1.25 | -1.97 – -0.54 | **0.001** |
| Observations | 379 | | | 378 | | | 379 | | | 378 | | | 379 | | | 378 | | |
| R^2^ / adjusted R^2^ | 0.157 / 0.155 | | | 0.352 / 0.347 | | | 0.187 / 0.185 | | | 0.496 / 0.490 | | | 0.355 / 0.349 | | | 0.512 / 0.504 | | |

**Table S4.** Emotional intelligence predicted from social class on 5-point scale and subjective inequality including covariates in Study 1a. Conservatism: 1= very liberal, 7 = very conservative; Gender coded as 1 = male, 0 = female. Conservatism, social class, unfairness beliefs, and subjective inequality are standardized.

|  | **Emotional Intelligence Model 1** | | | **Emotional Intelligence Model 2** | | | **Emotional Intelligence Model 3** | | | **Emotional Intelligence Model 4** | | | **Emotional Intelligence Model 5** | | | **Emotional Intelligence Model 6** | | |
| --- | --- | --- | --- | --- | --- | --- | --- | --- | --- | --- | --- | --- | --- | --- | --- | --- | --- | --- |
| *Predictors* | *Estimates* | *CI* | *p* | *Estimates* | *CI* | *p* | *Estimates* | *CI* | *p* | *Estimates* | *CI* | *p* | *Estimates* | *CI* | *p* | *Estimates* | *CI* | *p* |
| (Intercept) | 22.28 | 21.38 – 23.19 | **<0.001** | 24.81 | 23.63 – 26.00 | **<0.001** | 22.28 | 21.46 – 23.11 | **<0.001** | 24.08 | 23.02 – 25.15 | **<0.001** | 22.26 | 21.45 – 23.07 | **<0.001** | 24.05 | 22.98 – 25.12 | **<0.001** |
| Income | -1.04 | -1.94 – -0.13 | **0.025** | -0.70 | -1.48 – 0.08 | 0.080 |  |  |  | -0.83 | -1.52 – -0.13 | **0.020** | -1.28 | -2.10 – -0.46 | **0.002** | -0.88 | -1.58 – -0.18 | **0.014** |
| Conservatism |  |  |  | -3.80 | -4.59 – -3.02 | **<0.001** |  |  |  | -3.90 | -4.61 – -3.18 | **<0.001** |  |  |  | -3.84 | -4.56 – -3.12 | **<0.001** |
| Gender |  |  |  | -4.42 | -6.01 – -2.84 | **<0.001** |  |  |  | -3.12 | -4.55 – -1.69 | **<0.001** |  |  |  | -3.09 | -4.52 – -1.66 | **<0.001** |
| Subjective Inequality |  |  |  |  |  |  | -3.89 | -4.71 – -3.07 | **<0.001** | -3.24 | -4.11 – -2.37 | **<0.001** | -3.86 | -4.67 – -3.05 | **<0.001** | -3.24 | -4.11 – -2.38 | **<0.001** |
| Unfairness Beliefs |  |  |  |  |  |  |  |  |  | -0.55 | -1.42 – 0.32 | 0.213 |  |  |  | -0.50 | -1.38 – 0.37 | 0.259 |
| Income * Subjective Inequality |  |  |  |  |  |  |  |  |  |  |  |  | -0.93 | -1.72 – -0.14 | **0.021** | -0.41 | -1.09 – 0.27 | 0.236 |
| Observations | 379 | | | 378 | | | 379 | | | 378 | | | 379 | | | 378 | | |
| R^2^ / R^2^ adjusted | 0.013 / 0.011 | | | 0.277 / 0.271 | | | 0.187 / 0.185 | | | 0.431 / 0.423 | | | 0.215 / 0.208 | | | 0.433 / 0.424 | | |

**Table S5.** Emotional intelligence predicted from income and subjective inequality including covariates in Study 1a. Conservatism: 1= very liberal, 7 = very conservative; Gender coded as 1 = male, 0 = female. Income: 1=$15,000 or less, 2=$15,001-$25,000, 3=$25,001-$35,000, 4=$35,001-$50,000, 5=$50,001-$75,000, 6=$75,001-$100,000, 7=$100,001-$150,000, 8=$150,000 or more (measured as annual household income before tax). Income, conservatism, unfairness beliefs, and subjective inequality are standardized.

|  | **Emotional Intelligence Model 1** | | | **Emotional Intelligence Model 2** | | | **Emotional Intelligence Model 3** | | | **Emotional Intelligence Model 4** | | | **Emotional Intelligence Model 5** | | | **Emotional Intelligence Model 6** | | |
| --- | --- | --- | --- | --- | --- | --- | --- | --- | --- | --- | --- | --- | --- | --- | --- | --- | --- | --- |
| *Predictors* | *Estimates* | *CI* | *p* | *Estimates* | *CI* | *p* | *Estimates* | *CI* | *p* | *Estimates* | *CI* | *p* | *Estimates* | *CI* | *p* | *Estimates* | *CI* | *p* |
| (Intercept) | 22.33 | 21.48 – 23.17 | **<0.001** | 24.56 | 23.44 – 25.69 | **<0.001** | 22.31 | 21.49 – 23.13 | **<0.001** | 23.96 | 22.94 – 24.99 | **<0.001** | 22.39 | 21.59 – 23.19 | **<0.001** | 23.89 | 22.85 – 24.94 | **<0.001** |
| Years of Postsecondary Education | -3.31 | -4.16 – -2.46 | **<0.001** | -2.61 | -3.35 – -1.86 | **<0.001** |  |  |  | -2.08 | -2.77 – -1.39 | **<0.001** | -2.58 | -3.44 – -1.72 | **<0.001** | -2.19 | -2.93 – -1.45 | **<0.001** |
| Conservatism |  |  |  | -3.47 | -4.22 – -2.72 | **<0.001** |  |  |  | -3.63 | -4.33 – -2.93 | **<0.001** |  |  |  | -3.68 | -4.40 – -2.97 | **<0.001** |
| Gender |  |  |  | -3.94 | -5.45 – -2.44 | **<0.001** |  |  |  | -2.89 | -4.28 – -1.51 | **<0.001** |  |  |  | -2.88 | -4.26 – -1.50 | **<0.001** |
| Subjective Inequality |  |  |  |  |  |  | -3.86 | -4.68 – -3.03 | **<0.001** | -2.94 | -3.78 – -2.10 | **<0.001** | -3.47 | -4.29 – -2.65 | **<0.001** | -2.85 | -3.72 – -1.98 | **<0.001** |
| Unfairness Beliefs |  |  |  |  |  |  |  |  |  | -0.42 | -1.27 – 0.42 | 0.322 |  |  |  | -0.46 | -1.30 – 0.39 | 0.290 |
| Years of Postsecondary Education * Subjective Inequality |  |  |  |  |  |  |  |  |  |  |  |  | -0.43 | -1.51 – 0.65 | 0.433 | 0.37 | -0.57 – 1.32 | 0.437 |
| Observations | 378 | | | 377 | | | 378 | | | 377 | | | 378 | | | 377 | | |
| R^2^ / R^2^ adjusted | 0.136 / 0.134 | | | 0.349 / 0.343 | | | 0.184 / 0.182 | | | 0.469 / 0.462 | | | 0.274 / 0.268 | | | 0.470 / 0.461 | | |

**Table S6.** Emotional intelligence predicted from years of postsecondary education and subjective inequality including covariates in Study 1a. Conservatism: 1= very liberal, 7 = very conservative; Gender coded as 1 = male, 0 = female. Years of postsecondary education: Participants were asked to indicate how many years of postsecondary education they have taken. Education, conservatism, unfairness beliefs, and subjective inequality are standardized.

|  | **Emotional Intelligence Model 1** | | | **Emotional Intelligence Model 2** | | | **Emotional Intelligence Model 3** | | | **Emotional Intelligence Model 4** | | | **Emotional Intelligence Model 5** | | | **Emotional Intelligence Model 6** | | |
| --- | --- | --- | --- | --- | --- | --- | --- | --- | --- | --- | --- | --- | --- | --- | --- | --- | --- | --- |
| *Predictors* | *Estimates* | *CI* | *p* | *Estimates* | *CI* | *p* | *Estimates* | *CI* | *p* | *Estimates* | *CI* | *p* | *Estimates* | *CI* | *p* | *Estimates* | *CI* | *p* |
| (Intercept) | 22.33 | 21.48 – 23.17 | **<0.001** | 24.31 | 23.18 – 25.44 | **<0.001** | 22.31 | 21.49 – 23.13 | **<0.001** | 23.79 | 22.75 – 24.82 | **<0.001** | 22.61 | 21.83 – 23.39 | **<0.001** | 23.93 | 22.89 – 24.96 | **<0.001** |
| Highest Degree | -3.35 | -4.20 – -2.51 | **<0.001** | -2.66 | -3.41 – -1.91 | **<0.001** |  |  |  | -2.07 | -2.76 – -1.37 | **<0.001** | -2.71 | -3.50 – -1.93 | **<0.001** | -2.12 | -2.81 – -1.42 | **<0.001** |
| Conservatism |  |  |  | -3.60 | -4.34 – -2.85 | **<0.001** |  |  |  | -3.74 | -4.44 – -3.04 | **<0.001** |  |  |  | -3.58 | -4.29 – -2.87 | **<0.001** |
| Gender |  |  |  | -3.51 | -5.03 – -1.99 | **<0.001** |  |  |  | -2.58 | -3.98 – -1.19 | **<0.001** |  |  |  | -2.56 | -3.94 – -1.17 | **<0.001** |
| Subjective Inequality |  |  |  |  |  |  | -3.86 | -4.68 – -3.03 | **<0.001** | -2.91 | -3.75 – -2.07 | **<0.001** | -3.35 | -4.13 – -2.56 | **<0.001** | -3.00 | -3.84 – -2.16 | **<0.001** |
| Unfairness Beliefs |  |  |  |  |  |  |  |  |  | -0.40 | -1.24 – 0.44 | 0.351 |  |  |  | -0.29 | -1.13 – 0.55 | 0.500 |
| Highest Degree * Subjective Inequality |  |  |  |  |  |  |  |  |  |  |  |  | -1.43 | -2.18 – -0.67 | **<0.001** | -0.75 | -1.43 – -0.08 | **0.028** |
| Observations | 378 | | | 377 | | | 378 | | | 377 | | | 378 | | | 377 | | |
| R^2^ / R^2^ adjusted | 0.139 / 0.137 | | | 0.351 / 0.346 | | | 0.184 / 0.182 | | | 0.467 / 0.460 | | | 0.294 / 0.288 | | | 0.474 / 0.466 | | |

**Table S7.** Emotional intelligence predicted from highest degree and subjective inequality including covariates in Study 1a. Conservatism: 1= very liberal, 7 = very conservative; Gender coded as 1 = male, 0 = female. Degree: 1=no formal education, 2=elementary school, 3=junior high school, 4=senior high school or GED, 5=2-year college degree, 6=4-year college degree, 7=master's degree, 8=doctoral degree. Education, conservatism, unfairness beliefs, and subjective inequality are standardized.

|  | *Emotional Intelligence* | *Subjective Inequality* | *Subjective*  *Socioeconomic Status* | *Social Class* | *Income* | *Degree* | *Years of*  *Postsecondary Education* | *Unfairness*  *Beliefs* | *Conservatism* | *Age* | *Gender* |
| --- | --- | --- | --- | --- | --- | --- | --- | --- | --- | --- | --- |
| *Emotional*  *Intelligence* | .90 |  |  |  |  |  |  |  |  |  |  |
| *Subjective*  *Inequality* | -0.334 *(<.001)* | .89 |  |  |  |  |  |  |  |  |  |
| *Subjective*  *Socioeconomic Status* | -0.418 *(<.001)* | 0.064 *(.180)* | - |  |  |  |  |  |  |  |  |
| *Social*  *Class* | -0.291 *(<.001)* | -0.062 *(.194)* | 0.658 *(<.001)* | - |  |  |  |  |  |  |  |
| *Income* | 0.058 *(.227)* | -0.232 *(<.001)* | 0.378 *(<.001)* | 0.482 *(<.001)* | - |  |  |  |  |  |  |
| *Degree* | -0.191 *(<.001)* | 0.192 *(<.001)* | 0.377 *(<.001)* | 0.271 *(<.001)* | 0.187 *(<.001)* | - |  |  |  |  |  |
| *Years of*  *Postsecondary Education* | -0.302 *(<.001)* | 0.205 *(<.001)* | 0.221 *(<.001)* | 0.186 *(<.001)* | 0.042 *(.381)* | 0.379 *(<.001)* | - |  |  |  |  |
| *Unfairness*  *Beliefs* | -0.054 *(.263)* | 0.546 *(<.001)* | -0.021 *(.654)* | -0.051 *(.284)* | -0.134 *(.005)* | 0.165 *(.001)* | 0.150 *(.002)* | .81 |  |  |  |
| *Conservatism* | -0.471 *(<.001)* | -0.022 *(.647)* | 0.325 *(<.001)* | 0.233 *(<.001)* | 0.051 *(.290)* | 0.013 *(.783)* | 0.185 *(<.001)* | -0.227 *(<.001)* | - |  |  |
| *Age* | 0.209 *(<.001)* | -0.092 *(.054)* | -0.059 *(.216)* | -0.124 *(.009)* | -0.001 *(.978)* | 0.094 *(.050)* | 0.053 *(.268)* | -0.063 *(.189)* | 0.007 *(.878)* | - |  |
| *Gender* | -0.258 *(<.001)* | 0.069 *(.149)* | 0.134 *(.005)* | 0.103 *(.032)* | 0.025 *(.603)* | 0.114 *(.017)* | 0.008 *(.872)* | -0.071 *(.138)* | 0.131 *(.006)* | 0.017 *(.722)* | - |
| *Computed correlation used pearson-method with listwise-deletion.* | | | | | | | | | | | |

**Table S8.** Correlation between all measures (with Cronbach’s alpha on diagonal) in Study 1b. Income: 1=$15,000 or less, 2=$15,001-$25,000, 3=$25,001-$35,000, 4=$35,001-$50,000, 5=$50,001-$75,000, 6=$75,001-$100,000, 7=$100,001-$150,000, 8=$150,000 or more (measured as annual household income before tax); Degree: 1=no formal education, 2=elementary school, 3=junior high school, 4=senior high school or GED, 5=2-year college degree, 6=4-year college degree, 7=master's degree, 8=doctoral degree; Years of postsecondary education: Participants were asked to indicate how many years of postsecondary education they have taken; Conservatism: 1= very liberal, 7 = very conservative; Gender coded as 0 = female, 1 = male.


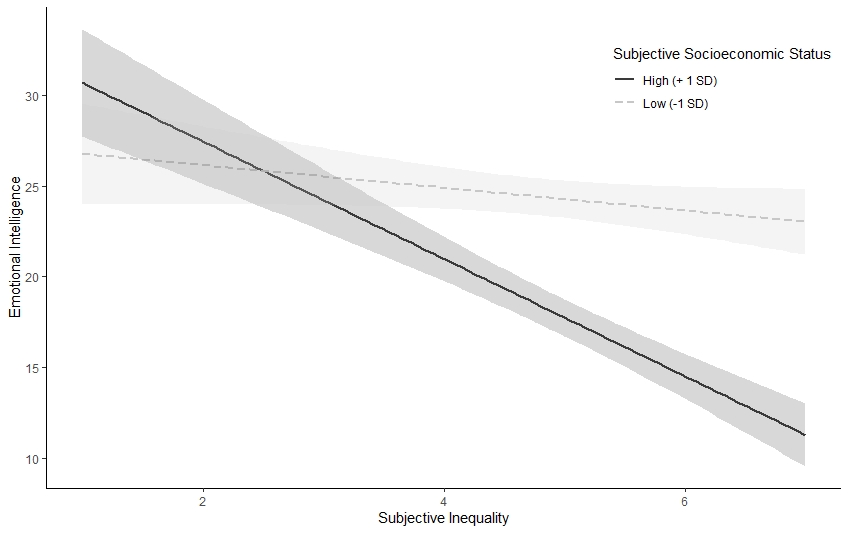


**Figure S2.** Association between subjective inequality and emotional intelligence at + 1SD (b=-4.36, p<.001) and at -1SD of SES (b=-1.91, p=0.003) in Study 1b. Intervals around regression lines are 95% confidence intervals.

|  | **Emotional Intelligence Model 1** | | | **Emotional Intelligence Model 2** | | | **Emotional Intelligence Model 3** | | | **Emotional Intelligence Model 4** | | | **Emotional Intelligence Model 5** | | | **Emotional Intelligence Model 6** | | |
| --- | --- | --- | --- | --- | --- | --- | --- | --- | --- | --- | --- | --- | --- | --- | --- | --- | --- | --- |
| *Predictors* | *Estimates* | *CI* | *p* | *Estimates* | *CI* | *p* | *Estimates* | *CI* | *p* | *Estimates* | *CI* | *p* | *Estimates* | *CI* | *p* | *Estimates* | *CI* | *p* |
| (Intercept) | 21.80 | 21.04 – 22.56 | **<0.001** | 23.65 | 22.56 – 24.73 | **<0.001** | 21.80 | 21.01 – 22.59 | **<0.001** | 23.40 | 22.39 – 24.41 | **<0.001** | 21.92 | 21.22 – 22.62 | **<0.001** | 23.48 | 22.49 – 24.47 | **<0.001** |
| Subjective SES | -3.74 | -4.51 – -2.98 | **<0.001** | -2.58 | -3.33 – -1.84 | **<0.001** |  |  |  | -2.32 | -3.01 – -1.63 | **<0.001** | -2.65 | -3.43 – -1.86 | **<0.001** | -1.70 | -2.43 – -0.96 | **<0.001** |
| Conservatism |  |  |  | -3.21 | -3.94 – -2.47 | **<0.001** |  |  |  | -3.30 | -4.00 – -2.59 | **<0.001** |  |  |  | -3.05 | -3.74 – -2.35 | **<0.001** |
| Gender |  |  |  | -3.16 | -4.58 – -1.73 | **<0.001** |  |  |  | -2.76 | -4.09 – -1.43 | **<0.001** |  |  |  | -2.69 | -3.99 – -1.38 | **<0.001** |
| Subjective Inequality |  |  |  |  |  |  | -2.99 | -3.78 – -2.20 | **<0.001** | -2.94 | -3.72 – -2.15 | **<0.001** | -2.82 | -3.52 – -2.12 | **<0.001** | -3.06 | -3.83 – -2.29 | **<0.001** |
| Unfairness Beliefs |  |  |  |  |  |  |  |  |  | 0.25 | -0.54 – 1.05 | 0.536 |  |  |  | 0.43 | -0.36 – 1.21 | 0.286 |
| Subjective SES* Subjective Inequality |  |  |  |  |  |  |  |  |  |  |  |  | -1.91 | -2.63 – -1.19 | **<0.001** | -1.51 | -2.19 – -0.83 | **<0.001** |
| Observations | 440 | | | 435 | | | 440 | | | 435 | | | 440 | | | 435 | | |
| R^2^ / adjusted R^2^ | 0.174 / 0.172 | | | 0.331 / 0.327 | | | 0.112 / 0.109 | | | 0.426 / 0.420 | | | 0.312 / 0.307 | | | 0.451 / 0.443 | | |

**Table S9.** Emotional intelligence predicted from SES on ladder scale and subjective inequality including covariates in Study 1b. Conservatism: 1= very liberal, 7 = very conservative; Gender coded as 1 = male, 0 = female; SES = socioeconomic status. Conservatism, SES, unfairness beliefs, and subjective inequality are standardized.

|  | **Emotional Intelligence Model 1** | | | **Emotional Intelligence Model 2** | | | **Emotional Intelligence Model 3** | | | **Emotional Intelligence Model 4** | | | **Emotional Intelligence Model 5** | | | **Emotional Intelligence Model 6** | | |
| --- | --- | --- | --- | --- | --- | --- | --- | --- | --- | --- | --- | --- | --- | --- | --- | --- | --- | --- |
| *Predictors* | *Estimates* | *CI* | *p* | *Estimates* | *CI* | *p* | *Estimates* | *CI* | *p* | *Estimates* | *CI* | *p* | *Estimates* | *CI* | *p* | *Estimates* | *CI* | *p* |
| (Intercept) | 21.80 | 21.00 – 22.60 | **<0.001** | 23.78 | 22.66 – 24.89 | **<0.001** | 21.80 | 21.01 – 22.59 | **<0.001** | 23.46 | 22.43 – 24.49 | **<0.001** | 21.71 | 20.97 – 22.44 | **<0.001** | 23.44 | 22.42 – 24.46 | **<0.001** |
| Social Class | -2.59 | -3.39 – -1.79 | **<0.001** | -1.70 | -2.44 – -0.95 | **<0.001** |  |  |  | -1.83 | -2.52 – -1.15 | **<0.001** | -2.29 | -3.06 – -1.51 | **<0.001** | -1.59 | -2.30 – -0.89 | **<0.001** |
| Conservatism |  |  |  | -3.63 | -4.37 – -2.89 | **<0.001** |  |  |  | -3.61 | -4.31 – -2.91 | **<0.001** |  |  |  | -3.44 | -4.14 – -2.74 | **<0.001** |
| Gender |  |  |  | -3.39 | -4.86 – -1.93 | **<0.001** |  |  |  | -2.88 | -4.23 – -1.52 | **<0.001** |  |  |  | -2.90 | -4.25 – -1.56 | **<0.001** |
| Subjective Inequality |  |  |  |  |  |  | -2.99 | -3.78 – -2.20 | **<0.001** | -3.22 | -4.02 – -2.43 | **<0.001** | -3.16 | -3.89 – -2.42 | **<0.001** | -3.21 | -4.00 – -2.42 | **<0.001** |
| Unfairness Beliefs |  |  |  |  |  |  |  |  |  | 0.29 | -0.52 – 1.10 | 0.486 |  |  |  | 0.29 | -0.51 – 1.10 | 0.477 |
| Social Class* Subjective Inequality |  |  |  |  |  |  |  |  |  |  |  |  | -1.49 | -2.20 – -0.78 | **<0.001** | -0.94 | -1.60 – -0.27 | **0.006** |
| Observations | 440 | | | 435 | | | 440 | | | 435 | | | 440 | | | 435 | | |
| R^2^ / adjusted R^2^ | 0.084 / 0.081 | | | 0.292 / 0.287 | | | 0.112 / 0.109 | | | 0.407 / 0.400 | | | 0.237 / 0.232 | | | 0.417 / 0.409 | | |

**Table S10.** Emotional intelligence predicted from social class on 5-point scale and subjective inequality including covariates in Study 1b. Conservatism: 1= very liberal, 7 = very conservative; Gender coded as 1 = male, 0 = female. Conservatism, social class, unfairness beliefs, and subjective inequality are standardized.

|  | **Emotional Intelligence Model 1** | | | **Emotional Intelligence Model 2** | | | **Emotional Intelligence Model 3** | | | **Emotional Intelligence Model 4** | | | **Emotional Intelligence Model 5** | | | **Emotional Intelligence Model 6** | | |
| --- | --- | --- | --- | --- | --- | --- | --- | --- | --- | --- | --- | --- | --- | --- | --- | --- | --- | --- |
| *Predictors* | *Estimates* | *CI* | *p* | *Estimates* | *CI* | *p* | *Estimates* | *CI* | *p* | *Estimates* | *CI* | *p* | *Estimates* | *CI* | *p* | *Estimates* | *CI* | *p* |
| (Intercept) | 21.80 | 20.96 – 22.64 | **<0.001** | 23.93 | 22.79 – 25.06 | **<0.001** | 21.80 | 21.01 – 22.59 | **<0.001** | 23.63 | 22.57 – 24.69 | **<0.001** | 21.56 | 20.75 – 22.37 | **<0.001** | 23.46 | 22.39 – 24.53 | **<0.001** |
| Income | 0.52 | -0.32 – 1.36 | 0.227 | 0.75 | 0.01 – 1.48 | **0.047** |  |  |  | 0.11 | -0.59 – 0.81 | 0.763 | -0.18 | -0.99 – 0.63 | 0.661 | 0.10 | -0.60 – 0.79 | 0.788 |
| Conservatism |  |  |  | -4.00 | -4.74 – -3.27 | **<0.001** |  |  |  | -4.01 | -4.72 – -3.31 | **<0.001** |  |  |  | -3.99 | -4.69 – -3.28 | **<0.001** |
| Gender |  |  |  | -3.68 | -5.17 – -2.19 | **<0.001** |  |  |  | -3.19 | -4.59 – -1.79 | **<0.001** |  |  |  | -3.16 | -4.55 – -1.76 | **<0.001** |
| Subjective Inequality |  |  |  |  |  |  | -2.99 | -3.79 – -2.20 | **<0.001** | -3.07 | -3.91 – -2.23 | **<0.001** | -3.03 | -3.84 – -2.22 | **<0.001** | -3.05 | -3.88 – -2.22 | **<0.001** |
| Unfairnes Beliefss |  |  |  |  |  |  |  |  |  | 0.17 | -0.67 – 1.01 | 0.688 |  |  |  | 0.15 | -0.68 – 0.98 | 0.724 |
| Income * Subjective Inequality |  |  |  |  |  |  |  |  |  |  |  |  | -1.02 | -1.77 – -0.27 | **0.008** | -0.71 | -1.38 – -0.04 | **0.036** |
| Observations | 440 | | | 435 | | | 440 | | | 435 | | | 440 | | | 435 | | |
| R^2^ / R^2^ adjusted | 0.003 / 0.001 | | | 0.266 / 0.261 | | | 0.112 / 0.109 | | | 0.369 / 0.362 | | | 0.126 / 0.120 | | | 0.375 / 0.367 | | |

**Table S11.** Emotional intelligence predicted from income and subjective inequality including covariates in Study 1b. Conservatism: 1= very liberal, 7 = very conservative; Gender coded as 1 = male, 0 = female. Income: 1=$15,000 or less, 2=$15,001-$25,000, 3=$25,001-$35,000, 4=$35,001-$50,000, 5=$50,001-$75,000, 6=$75,001-$100,000, 7=$100,001-$150,000, 8=$150,000 or more (measured as annual household income before tax). Income, conservatism, unfairness beliefs, and subjective inequality are standardized.

|  | **Emotional Intelligence Model 1** | | | **Emotional Intelligence Model 2** | | | **Emotional Intelligence Model 3** | | | **Emotional Intelligence Model 4** | | | **Emotional Intelligence Model 5** | | | **Emotional Intelligence Model 6** | | |
| --- | --- | --- | --- | --- | --- | --- | --- | --- | --- | --- | --- | --- | --- | --- | --- | --- | --- | --- |
| *Predictors* | *Estimates* | *CI* | *p* | *Estimates* | *CI* | *p* | *Estimates* | *CI* | *p* | *Estimates* | *CI* | *p* | *Estimates* | *CI* | *p* | *Estimates* | *CI* | *p* |
| (Intercept) | 21.80 | 21.00 – 22.60 | **<0.001** | 23.96 | 22.86 – 25.07 | **<0.001** | 21.80 | 21.01 – 22.59 | **<0.001** | 23.67 | 22.63 – 24.72 | **<0.001** | 21.85 | 21.07 – 22.64 | **<0.001** | 23.67 | 22.61 – 24.73 | **<0.001** |
| Years of Postsecondary Education | -2.71 | -3.51 – -1.91 | **<0.001** | -2.02 | -2.74 – -1.31 | **<0.001** |  |  |  | -1.46 | -2.15 – -0.77 | **<0.001** | -2.10 | -2.94 – -1.26 | **<0.001** | -1.46 | -2.20 – -0.73 | **<0.001** |
| Conservatism |  |  |  | -3.61 | -4.34 – -2.88 | **<0.001** |  |  |  | -3.70 | -4.41 – -2.99 | **<0.001** |  |  |  | -3.70 | -4.41 – -2.99 | **<0.001** |
| Gender |  |  |  | -3.71 | -5.16 – -2.27 | **<0.001** |  |  |  | -3.25 | -4.62 – -1.88 | **<0.001** |  |  |  | -3.25 | -4.62 – -1.87 | **<0.001** |
| Subjective Inequality |  |  |  |  |  |  | -2.99 | -3.79 – -2.20 | **<0.001** | -2.87 | -3.68 – -2.06 | **<0.001** | -2.57 | -3.36 – -1.78 | **<0.001** | -2.87 | -3.68 – -2.05 | **<0.001** |
| Unfairness Beliefs |  |  |  |  |  |  |  |  |  | 0.35 | -0.48 – 1.17 | 0.410 |  |  |  | 0.35 | -0.48 – 1.17 | 0.411 |
| Years of Postsecondary Education * Subjective Inequality |  |  |  |  |  |  |  |  |  |  |  |  | -0.27 | -1.10 – 0.56 | 0.524 | 0.02 | -0.70 – 0.74 | 0.966 |
| Observations | 440 | | | 435 | | | 440 | | | 435 | | | 440 | | | 435 | | |
| R^2^ / R^2^ adjusted | 0.092 / 0.090 | | | 0.308 / 0.303 | | | 0.112 / 0.109 | | | 0.393 / 0.386 | | | 0.170 / 0.164 | | | 0.393 / 0.384 | | |

**Table S12.** Emotional intelligence predicted from years of postsecondary education and subjective inequality including covariates in Study 1b. Conservatism: 1= very liberal, 7 = very conservative; Gender coded as 1 = male, 0 = female. Years of postsecondary education: Participants were asked to indicate how many years of postsecondary education they have taken. Education, conservatism, unfairness beliefs, and subjective inequality are standardized.

|  | **Emotional Intelligence Model 1** | | | **Emotional Intelligence Model 2** | | | **Emotional Intelligence Model 3** | | | **Emotional Intelligence Model 4** | | | **Emotional Intelligence Model 5** | | | **Emotional Intelligence Model 6** | | |
| --- | --- | --- | --- | --- | --- | --- | --- | --- | --- | --- | --- | --- | --- | --- | --- | --- | --- | --- |
| *Predictors* | *Estimates* | *CI* | *p* | *Estimates* | *CI* | *p* | *Estimates* | *CI* | *p* | *Estimates* | *CI* | *p* | *Estimates* | *CI* | *p* | *Estimates* | *CI* | *p* |
| (Intercept) | 21.80 | 20.97 – 22.62 | **<0.001** | 23.72 | 22.60 – 24.85 | **<0.001** | 21.80 | 21.01 – 22.59 | **<0.001** | 23.51 | 22.45 – 24.57 | **<0.001** | 21.99 | 21.20 – 22.78 | **<0.001** | 23.60 | 22.54 – 24.66 | **<0.001** |
| Highest Degree | -1.72 | -2.55 – -0.89 | **<0.001** | -1.50 | -2.22 – -0.77 | **<0.001** |  |  |  | -0.96 | -1.66 – -0.27 | **0.007** | -1.35 | -2.16 – -0.55 | **0.001** | -1.06 | -1.77 – -0.36 | **0.003** |
| Conservatism |  |  |  | -3.99 | -4.72 – -3.27 | **<0.001** |  |  |  | -3.99 | -4.69 – -3.29 | **<0.001** |  |  |  | -3.93 | -4.63 – -3.23 | **<0.001** |
| Gender |  |  |  | -3.30 | -4.78 – -1.82 | **<0.001** |  |  |  | -2.97 | -4.36 – -1.57 | **<0.001** |  |  |  | -2.94 | -4.33 – -1.55 | **<0.001** |
| Subjective Inequality |  |  |  |  |  |  | -2.99 | -3.79 – -2.20 | **<0.001** | -2.96 | -3.78 – -2.14 | **<0.001** | -2.81 | -3.61 – -2.01 | **<0.001** | -2.99 | -3.81 – -2.17 | **<0.001** |
| Unfairness Beliefs |  |  |  |  |  |  |  |  |  | 0.28 | -0.55 – 1.11 | 0.507 |  |  |  | 0.30 | -0.53 – 1.14 | 0.474 |
| Highest Degree * Subjective Inequality |  |  |  |  |  |  |  |  |  |  |  |  | -1.03 | -1.77 – -0.28 | **0.007** | -0.53 | -1.18 – 0.13 | 0.115 |
| Observations | 440 | | | 435 | | | 440 | | | 435 | | | 440 | | | 435 | | |
| R^2^ / R^2^ adjusted | 0.037 / 0.035 | | | 0.286 / 0.281 | | | 0.112 / 0.109 | | | 0.379 / 0.372 | | | 0.143 / 0.137 | | | 0.383 / 0.374 | | |

**Table S13.** Emotional intelligence predicted from highest degree and subjective inequality including covariates in Study 1b. Conservatism: 1= very liberal, 7 = very conservative; Gender coded as 1 = male, 0 = female. Degree: 1=no formal education, 2=elementary school, 3=junior high school, 4=senior high school or GED, 5=2-year college degree, 6=4-year college degree, 7=master's degree, 8=doctoral degree. Education, conservatism, unfairness beliefs, and subjective inequality are standardized.

|  | *Emotional Intelligence* | *Subjective Inequality* | *Subjective*  *Socioeconomic Status* | *Income* | *Degree* | *Years of*  *Postsecondary Education* | *Unfairness*  *Beliefs* | *Conservatism* | *Age* | *Gender* |
| --- | --- | --- | --- | --- | --- | --- | --- | --- | --- | --- |
| *Emotional*  *Intelligence* | .91 |  |  |  |  |  |  |  |  |  |
| *Subjective*  *Inequality* | -0.306 *(<.001)* | .91 |  |  |  |  |  |  |  |  |
| *Subjective*  *Socioeconomic*  *Status* | -0.369 *(<.001)* | -0.017 *(.791)* | - |  |  |  |  |  |  |  |
| *Income* | -0.078 *(.220)* | -0.169 *(.008)* | 0.453 *(<.001)* | - |  |  |  |  |  |  |
| *Degree* | -0.236 *(<.001)* | 0.102 *(.107)* | 0.367 *(<.001)* | 0.378 *(<.001)* | - |  |  |  |  |  |
| *Years of*  *Postsecondary Education* | -0.269 *(<.001)* | 0.135 *(.032)* | 0.219 *(<.001)* | 0.111 *(.080)* | 0.410 *(<.001)* | - |  |  |  |  |
| *Unfairness*  *Beliefs* | -0.042 *(.505)* | 0.564 *(<.001)* | -0.069 *(.279)* | -0.206 *(.001)* | -0.015 *(.810)* | -0.002 *(.975)* | .86 |  |  |  |
| *Conservatism* | -0.307 *(<.001)* | -0.065 *(.304)* | 0.172 *(.006)* | 0.136 *(.032)* | 0.067 *(.288)* | 0.105 *(.099)* | -0.316 *(<.001)* | - |  |  |
| *Age* | 0.339 *(<.001)* | -0.142 *(.024)* | -0.182 *(.004)* | -0.048 *(.447)* | -0.035 *(.577)* | -0.007 *(.918)* | -0.016 *(.807)* | 0.006 *(.925)* | - |  |
| *Gender* | -0.262 *(<.001)* | -0.055 *(.385)* | 0.148 *(.019)* | 0.001 *(.984)* | 0.060 *(.350)* | -0.010 *(.875)* | -0.167 *(.008)* | 0.097 *(.128)* | -0.136 *(.031)* | - |
| *Computed correlation used pearson-method with listwise-deletion.* | | | | | | | | | | |

**Table S14.** Correlation between all measures (with Cronbach’s alpha on diagonal) in Study 2a. Income: 1=less than $10,000, 2=$10,00-$19,999, 3-11 in $10,000 increments, 12=$110,000-$119,999, 13=More than $120,000 (measured as annual household income); Degree: 1=no schooling completed, 2=nursery school to 8^th^ grade, 3=some high school, no diploma, 4=high school graduate, diploma or the equivalent (e.g., GED), 5=some college credit, no degree, 6=trade/technical/vocational training, 7=associate degree, 8=bachelor’s degree, 9=master's degree, 10=professional degree, 11=doctoral degree; Years of postsecondary education: Participants were asked to indicate how many years of postsecondary education they have taken. Conservatism: 1= very liberal, 7 = very conservative; Gender coded as 0 = female, 1 = male.

**
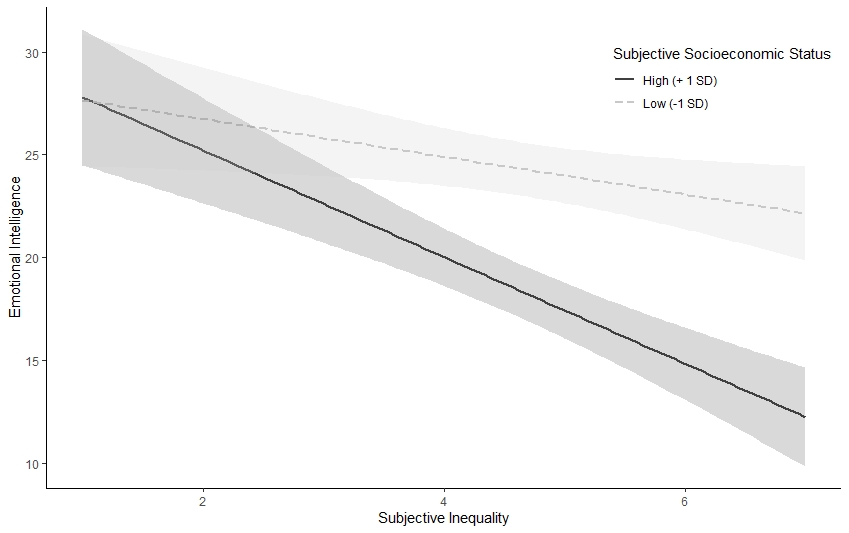
**

**Figure S3.** Association between subjective inequality and emotional intelligence at + 1SD (b=-3.90, p<.001) and at -1SD of SES (b=-1.38, p=0.026) in Study 2a. Intervals around regression lines are 95% confidence intervals.

|  | **Emotional Intelligence Model 1** | | | **Emotional Intelligence Model 2** | | | **Emotional Intelligence Model 3** | | | **Emotional Intelligence Model 4** | | | **Emotional Intelligence Model 5** | | | **Emotional Intelligence Model 6** | | |
| --- | --- | --- | --- | --- | --- | --- | --- | --- | --- | --- | --- | --- | --- | --- | --- | --- | --- | --- |
| *Predictors* | *Estimates* | *CI* | *p* | *Estimates* | *CI* | *p* | *Estimates* | *CI* | *p* | *Estimates* | *CI* | *p* | *Estimates* | *CI* | *p* | *Estimates* | *CI* | *p* |
| (Intercept) | 21.72 | 20.74 – 22.69 | **<0.001** | 23.50 | 22.14 – 24.85 | **<0.001** | 21.36 | 20.41 – 22.31 | **<0.001** | 23.60 | 22.34 – 24.86 | **<0.001** | 21.69 | 20.78 – 22.60 | **<0.001** | 23.58 | 22.34 – 24.81 | **<0.001** |
| Subjective SES | -3.11 | -4.08 – -2.13 | **<0.001** | -2.54 | -3.49 – -1.59 | **<0.001** |  |  |  | -2.55 | -3.44 – -1.67 | **<0.001** | -2.81 | -3.75 – -1.87 | **<0.001** | -2.23 | -3.12 – -1.34 | **<0.001** |
| Conservatism |  |  |  | -1.88 | -2.82 – -0.93 | **<0.001** |  |  |  | -1.96 | -2.88 – -1.03 | **<0.001** |  |  |  | -1.84 | -2.75 – -0.92 | **<0.001** |
| Gender |  |  |  | -3.56 | -5.44 – -1.68 | **<0.001** |  |  |  | -3.78 | -5.55 – -2.02 | **<0.001** |  |  |  | -3.77 | -5.51 – -2.04 | **<0.001** |
| Subjective Inequality |  |  |  |  |  |  | -2.79 | -3.74 – -1.84 | **<0.001** | -2.98 | -4.01 – -1.95 | **<0.001** | -2.64 | -3.54 – -1.74 | **<0.001** | -3.14 | -4.15 – -2.12 | **<0.001** |
| Unfairness Beliefs |  |  |  |  |  |  |  |  |  | 0.30 | -0.77 – 1.36 | 0.585 |  |  |  | 0.45 | -0.60 – 1.51 | 0.399 |
| Subjective SES* Subjective Inequality |  |  |  |  |  |  |  |  |  |  |  |  | -1.26 | -2.10 – -0.42 | **0.004** | -1.22 | -2.01 – -0.44 | **0.002** |
| Observations | 253 | | | 252 | | | 282 | | | 252 | | | 253 | | | 252 | | |
| R^2^ / adjusted R^2^ | 0.134 / 0.130 | | | 0.232 / 0.222 | | | 0.106 / 0.103 | | | 0.345 / 0.331 | | | 0.253 / 0.244 | | | 0.369 / 0.353 | | |

**Table S15.** Emotional intelligence predicted from SES and subjective inequality including covariates in Study 2a. Conservatism: 1= very liberal, 7 = very conservative; Gender coded as 1 = male, 0 = female; SES = socioeconomic status. Conservatism, SES, unfairness beliefs, and subjective inequality are standardized.

|  | **Emotional Intelligence Model 1** | | | **Emotional Intelligence Model 2** | | | **Emotional Intelligence Model 3** | | | **Emotional Intelligence Model 4** | | | **Emotional Intelligence Model 5** | | | **Emotional Intelligence Model 6** | | |
| --- | --- | --- | --- | --- | --- | --- | --- | --- | --- | --- | --- | --- | --- | --- | --- | --- | --- | --- |
| *Predictors* | *Estimates* | *CI* | *p* | *Estimates* | *CI* | *p* | *Estimates* | *CI* | *p* | *Estimates* | *CI* | *p* | *Estimates* | *CI* | *p* | *Estimates* | *CI* | *p* |
| (Intercept) | 21.29 | 20.28 – 22.30 | **<0.001** | 23.37 | 21.99 – 24.75 | **<0.001** | 21.36 | 20.41 – 22.31 | **<0.001** | 23.42 | 22.14 – 24.70 | **<0.001** | 21.07 | 20.13 – 22.02 | **<0.001** | 23.31 | 22.06 – 24.57 | **<0.001** |
| Income | -0.63 | -1.64 – 0.38 | 0.223 | -0.36 | -1.31 – 0.59 | 0.455 |  |  |  | -0.84 | -1.74 – 0.05 | 0.064 | -1.10 | -2.05 – -0.15 | **0.023** | -0.83 | -1.70 – 0.05 | 0.063 |
| Conservatism |  |  |  | -2.26 | -3.22 – -1.31 | **<0.001** |  |  |  | -2.26 | -3.18 – -1.34 | **<0.001** |  |  |  | -2.18 | -3.08 – -1.27 | **<0.001** |
| Gender |  |  |  | -3.97 | -5.87 – -2.07 | **<0.001** |  |  |  | -4.08 | -5.86 – -2.31 | **<0.001** |  |  |  | -4.34 | -6.08 – -2.60 | **<0.001** |
| Subjective Inequality |  |  |  |  |  |  | -2.79 | -3.74 – -1.84 | **<0.001** | -3.34 | -4.41 – -2.27 | **<0.001** | -2.95 | -3.90 – -2.01 | **<0.001** | -3.37 | -4.41 – -2.32 | **<0.001** |
| Unfairness Beliefs |  |  |  |  |  |  |  |  |  | 0.32 | -0.80 – 1.45 | 0.572 |  |  |  | 0.46 | -0.64 – 1.57 | 0.408 |
| Income * Subjective Inequality |  |  |  |  |  |  |  |  |  |  |  |  | -1.35 | -2.24 – -0.46 | **0.003** | -1.52 | -2.34 – -0.71 | **<0.001** |
| Observations | 279 | | | 278 | | | 282 | | | 278 | | | 279 | | | 278 | | |
| R^2^ / R^2^ adjusted | 0.005 / 0.002 | | | 0.138 / 0.129 | | | 0.106 / 0.103 | | | 0.273 / 0.259 | | | 0.153 / 0.144 | | | 0.307 / 0.292 | | |

**Table S16.** Emotional intelligence predicted from income and subjective inequality including covariates in Study 2a. Conservatism: 1= very liberal, 7 = very conservative; Gender coded as 1 = male, 0 = female. Income: 1=less than $10,000, 2=$10,00-$19,999, 3-11 in $10,000 increments, 12=$110,000-$119,999, 13=More than $120,000 (measured as annual household income). Income, conservatism, unfairness beliefs, and subjective inequality are standardized.

|  | **Emotional Intelligence Model 1** | | | **Emotional Intelligence Model 2** | | | **Emotional Intelligence Model 3** | | | **Emotional Intelligence Model 4** | | | **Emotional Intelligence Model 5** | | | **Emotional Intelligence Model 6** | | |
| --- | --- | --- | --- | --- | --- | --- | --- | --- | --- | --- | --- | --- | --- | --- | --- | --- | --- | --- |
| *Predictors* | *Estimates* | *CI* | *p* | *Estimates* | *CI* | *p* | *Estimates* | *CI* | *p* | *Estimates* | *CI* | *p* | *Estimates* | *CI* | *p* | *Estimates* | *CI* | *p* |
| (Intercept) | 21.40 | 20.43 – 22.37 | **<0.001** | 23.50 | 22.19 – 24.82 | **<0.001** | 21.36 | 20.41 – 22.31 | **<0.001** | 23.56 | 22.33 – 24.80 | **<0.001** | 21.36 | 20.42 – 22.30 | **<0.001** | 23.51 | 22.26 – 24.77 | **<0.001** |
| Years of Postsecondary Education | -2.38 | -3.35 – -1.41 | **<0.001** | -2.25 | -3.15 – -1.34 | **<0.001** |  |  |  | -1.84 | -2.70 – -0.98 | **<0.001** | -2.05 | -2.99 – -1.10 | **<0.001** | -1.87 | -2.74 – -1.00 | **<0.001** |
| Conservatism |  |  |  | -2.15 | -3.06 – -1.24 | **<0.001** |  |  |  | -2.27 | -3.16 – -1.37 | **<0.001** |  |  |  | -2.29 | -3.19 – -1.39 | **<0.001** |
| Gender |  |  |  | -4.01 | -5.82 – -2.19 | **<0.001** |  |  |  | -4.15 | -5.87 – -2.44 | **<0.001** |  |  |  | -4.13 | -5.85 – -2.40 | **<0.001** |
| Subjective Inequality |  |  |  |  |  |  | -2.79 | -3.74 – -1.84 | **<0.001** | -2.80 | -3.86 – -1.75 | **<0.001** | -2.51 | -3.45 – -1.56 | **<0.001** | -2.75 | -3.83 – -1.67 | **<0.001** |
| Unfairness Beliefs |  |  |  |  |  |  |  |  |  | 0.08 | -1.03 – 1.19 | 0.886 |  |  |  | 0.05 | -1.07 – 1.17 | 0.930 |
| Years of Postsecondary Education * Subjective Inequality |  |  |  |  |  |  |  |  |  |  |  |  | 0.26 | -0.91 – 1.43 | 0.664 | 0.27 | -0.81 – 1.36 | 0.622 |
| Observations | 279 | | | 278 | | | 282 | | | 278 | | | 279 | | | 278 | | |
| R^2^ / R^2^ adjusted | 0.078 / 0.075 | | | 0.206 / 0.197 | | | 0.106 / 0.103 | | | 0.309 / 0.296 | | | 0.166 / 0.157 | | | 0.309 / 0.294 | | |

**Table S17.** Emotional intelligence predicted from years of postsecondary education and subjective inequality including covariates in Study 2a. Conservatism: 1= very liberal, 7 = very conservative; Gender coded as 1 = male, 0 = female. Years of postsecondary education: Participants were asked to indicate how many years of postsecondary education they have taken. Education, conservatism, unfairness beliefs, and subjective inequality are standardized.

|  | **Emotional Intelligence Model 1** | | | **Emotional Intelligence Model 2** | | | **Emotional Intelligence Model 3** | | | **Emotional Intelligence Model 4** | | | **Emotional Intelligence Model 5** | | | **Emotional Intelligence Model 6** | | |
| --- | --- | --- | --- | --- | --- | --- | --- | --- | --- | --- | --- | --- | --- | --- | --- | --- | --- | --- |
| *Predictors* | *Estimates* | *CI* | *p* | *Estimates* | *CI* | *p* | *Estimates* | *CI* | *p* | *Estimates* | *CI* | *p* | *Estimates* | *CI* | *p* | *Estimates* | *CI* | *p* |
| (Intercept) | 21.36 | 20.38 – 22.34 | **<0.001** | 23.40 | 22.06 – 24.75 | **<0.001** | 21.36 | 20.41 – 22.31 | **<0.001** | 23.49 | 22.22 – 24.76 | **<0.001** | 21.42 | 20.47 – 22.36 | **<0.001** | 23.54 | 22.27 – 24.82 | **<0.001** |
| Highest Degree | -1.83 | -2.81 – -0.84 | **<0.001** | -1.57 | -2.50 – -0.64 | **0.001** |  |  |  | -1.23 | -2.11 – -0.36 | **0.006** | -1.53 | -2.47 – -0.58 | **0.002** | -1.23 | -2.11 – -0.36 | **0.006** |
| Conservatism |  |  |  | -2.15 | -3.07 – -1.22 | **<0.001** |  |  |  | -2.19 | -3.11 – -1.28 | **<0.001** |  |  |  | -2.12 | -3.05 – -1.18 | **<0.001** |
| Gender |  |  |  | -3.92 | -5.78 – -2.06 | **<0.001** |  |  |  | -4.10 | -5.86 – -2.34 | **<0.001** |  |  |  | -4.12 | -5.88 – -2.36 | **<0.001** |
| Subjective Inequality |  |  |  |  |  |  | -2.79 | -3.74 – -1.84 | **<0.001** | -3.03 | -4.09 – -1.97 | **<0.001** | -2.59 | -3.53 – -1.64 | **<0.001** | -3.06 | -4.13 – -1.99 | **<0.001** |
| Unfairness Beliefs |  |  |  |  |  |  |  |  |  | 0.32 | -0.79 – 1.44 | 0.565 |  |  |  | 0.42 | -0.72 – 1.55 | 0.470 |
| Highest Degree * Subjective Inequality |  |  |  |  |  |  |  |  |  |  |  |  | -0.52 | -1.51 – 0.48 | 0.309 | -0.37 | -1.32 – 0.58 | 0.445 |
| Observations | 282 | | | 281 | | | 282 | | | 281 | | | 282 | | | 281 | | |
| R^2^ / R^2^ adjusted | 0.045 / 0.042 | | | 0.169 / 0.160 | | | 0.106 / 0.103 | | | 0.279 / 0.266 | | | 0.141 / 0.132 | | | 0.281 / 0.265 | | |

**Table S18.** Emotional intelligence predicted from highest degree and subjective inequality including covariates in Study 2a. Conservatism: 1= very liberal, 7 = very conservative; Gender coded as 1 = male, 0 = female. Degree: 1=no schooling completed, 2=nursery school to 8^th^ grade, 3=some high school, no diploma, 4=high school graduate, diploma or the equivalent (e.g., GED), 5=some college credit, no degree, 6=trade/technical/vocational training, 7=associate degree, 8=bachelor’s degree, 9=master's degree, 10=professional degree, 11=doctoral degree. Education, conservatism, unfairness beliefs, and subjective inequality are standardized.

|  | *Emotional Intelligence* | *Subjective Inequality* | *Subjective*  *Socioeconomic Status* | *Income* | *Degree* | *Years of*  *Postsecondary Education* | *Unfairness*  *Beliefs* | *Conservatism* | *Age* | *Gender* |
| --- | --- | --- | --- | --- | --- | --- | --- | --- | --- | --- |
| *Emotional*  *Intelligence* | .66 |  |  |  |  |  |  |  |  |  |
| *Subjective*  *Inequality* | -0.036 *(.482)* | .78 |  |  |  |  |  |  |  |  |
| *Subjective*  *Socioeconomic*  *Status* | 0.034 *(.504)* | -0.072 *(.154)* | - |  |  |  |  |  |  |  |
| *Income* | -0.128 *(.011)* | 0.175 *(.001)* | -0.419 *(<.001)* | - |  |  |  |  |  |  |
| *Degree* | 0.022 *(.661)* | 0.017 *(.736)* | 0.183 *(<.001)* | -0.145 *(.004)* | - |  |  |  |  |  |
| *Years of*  *Postsecondary Education* | -0.009 *(.858)* | 0.060 *(.237)* | 0.137 *(.007)* | -0.044 *(.392)* | 0.685 *(<.001)* | - |  |  |  |  |
| *Unfairness*  *Beliefs* | 0.138 *(.007)* | 0.461 *(<.001)* | -0.081 *(.111)* | 0.083 *(.103)* | 0.055 *(.277)* | 0.052 *(.304)* | .76 |  |  |  |
| *Conservatism* | -0.188 *(<.001)* | -0.293 *(<.001)* | -0.047 *(.358)* | 0.015 *(.766)* | -0.080 *(.117)* | -0.102 *(.046)* | -0.346 *(<.001)* | - |  |  |
| *Age* | 0.044 *(.392)* | -0.244 *(<.001)* | 0.098 *(.055)* | -0.244 *(<.001)* | 0.363 *(<.001)* | 0.220 *(<.001)* | -0.110 *(.030)* | 0.099 *(.052)* | - |  |
| *Gender* | -0.146 *(.004)* | -0.052 *(.310)* | 0.070 *(.171)* | -0.027 *(.599)* | 0.042 *(.414)* | 0.009 *(.859)* | -0.113 *(.027)* | 0.08831 *(.106)* | 0.015 *(.776)* | - |
| *Computed correlation used pearson-method with listwise-deletion.* | | | | | | | | | | |

**Table S19.** Correlation between all measures (with Cronbach’s alpha on diagonal) in Study 2b. Income: 1=$20,000 or less, 2=$20,00-$39,999, 3=$40,00-$59,999, 4=$60,00-$79,999, 5=$80,000-$99,999, 6=$100,000-$119,999, 7=$120,000-$159,999, 8=$160,000-$199,999, 9=$200,000-$299,999, 10=$300,000-$399,999, 11=$400,000 or above (measured as annual household income before tax); Degree: 1=no formal education, 2=elementary school, 3=junior high school, 4=senior high school or GED, 5=2-year college degree, 6=4-year college degree, 7=master's degree, 8=doctoral degree; Years of postsecondary education: Participants were asked to indicate how many years of postsecondary education they have taken. Conservatism: 1= very liberal, 7 = very conservative; Gender coded as 0 = female, 1 = male.

|  | **Emotional Intelligence Model 1** | | | **Emotional Intelligence Model 2** | | | **Emotional Intelligence Model 3** | | | **Emotional Intelligence Model 4** | | | **Emotional Intelligence Model 5** | | | **Emotional Intelligence Model 6** | | |
| --- | --- | --- | --- | --- | --- | --- | --- | --- | --- | --- | --- | --- | --- | --- | --- | --- | --- | --- |
| *Predictors* | *Estimates* | *CI* | *p* | *Estimates* | *CI* | *p* | *Estimates* | *CI* | *p* | *Estimates* | *CI* | *p* | *Estimates* | *CI* | *p* | *Estimates* | *CI* | *p* |
| (Intercept) | 25.73 | 25.30 – 26.17 | **<0.001** | 26.30 | 25.71 – 26.88 | **<0.001** | 25.73 | 25.30 – 26.17 | **<0.001** | 26.26 | 25.69 – 26.84 | **<0.001** | 25.73 | 25.29 – 26.16 | **<0.001** | 26.26 | 25.68 – 26.84 | **<0.001** |
| Subjective SES | 0.19 | -0.25 – 0.62 | 0.395 | 0.19 | -0.25 – 0.62 | 0.399 |  |  |  | 0.19 | -0.25 – 0.62 | 0.400 | 0.17 | -0.27 – 0.61 | 0.439 | 0.18 | -0.25 – 0.61 | 0.412 |
| Conservatism |  |  |  | -0.77 | -1.20 – -0.34 | **<0.001** |  |  |  | -0.75 | -1.21 – -0.29 | **0.001** |  |  |  | -0.75 | -1.21 – -0.29 | **0.001** |
| Gender |  |  |  | -1.18 | -2.04 – -0.32 | **0.007** |  |  |  | -1.12 | -1.98 – -0.26 | **0.011** |  |  |  | -1.12 | -1.98 – -0.26 | **0.011** |
| Subjective Inequality |  |  |  |  |  |  | -0.12 | -0.56 – 0.31 | 0.586 | -0.60 | -1.08 – -0.12 | **0.014** | -0.11 | -0.55 – 0.32 | 0.616 | -0.60 | -1.08 – -0.12 | **0.014** |
| Unfairness Beliefs |  |  |  |  |  |  |  |  |  | 0.55 | 0.05 – 1.05 | **0.030** |  |  |  | 0.55 | 0.05 – 1.05 | **0.031** |
| Subjective SES* Subjective Inequality |  |  |  |  |  |  |  |  |  |  |  |  | -0.09 | -0.53 – 0.35 | 0.696 | -0.04 | -0.47 – 0.40 | 0.869 |
| Observations | 406 | | | 401 | | | 406 | | | 401 | | | 406 | | | 401 | | |
| R^2^ / adjusted R^2^ | 0.002 / -0.001 | | | 0.054 / 0.046 | | | 0.001 / -0.002 | | | 0.072 / 0.060 | | | 0.003 / -0.005 | | | 0.072 / 0.058 | | |

**Table S20.** Emotional intelligence predicted from SES and subjective inequality with covariates in Study 2b. Conservatism: 1= very liberal, 7 = very conservative; Gender coded as 1 = male, 0 = female; SES = socioeconomic status. Conservatism, SES, unfairness beliefs, and subjective inequality are standardized.

|  | **Emotional Intelligence Model 1** | | | **Emotional Intelligence Model 2** | | | **Emotional Intelligence Model 3** | | | **Emotional Intelligence Model 4** | | | **Emotional Intelligence Model 5** | | | **Emotional Intelligence Model 6** | | |
| --- | --- | --- | --- | --- | --- | --- | --- | --- | --- | --- | --- | --- | --- | --- | --- | --- | --- | --- |
| *Predictors* | *Estimates* | *CI* | *p* | *Estimates* | *CI* | *p* | *Estimates* | *CI* | *p* | *Estimates* | *CI* | *p* | *Estimates* | *CI* | *p* | *Estimates* | *CI* | *p* |
| (Intercept) | 25.75 | 25.31 – 26.18 | **<0.001** | 26.31 | 25.73 – 26.90 | **<0.001** | 25.73 | 25.30 – 26.17 | **<0.001** | 26.28 | 25.69 – 26.86 | **<0.001** | 25.76 | 25.32 – 26.21 | **<0.001** | 26.29 | 25.70 – 26.87 | **<0.001** |
| Income | -0.55 | -0.99 – -0.11 | **0.014** | -0.53 | -0.95 – -0.10 | **0.016** |  |  |  | -0.49 | -0.92 – -0.06 | **0.027** | -0.55 | -1.00 – -0.11 | **0.015** | -0.49 | -0.93 – -0.06 | **0.027** |
| Conservatism |  |  |  | -0.75 | -1.18 – -0.32 | **0.001** |  |  |  | -0.71 | -1.17 – -0.25 | **0.003** |  |  |  | -0.71 | -1.17 – -0.25 | **0.003** |
| Gender |  |  |  | -1.20 | -2.06 – -0.33 | **0.007** |  |  |  | -1.13 | -1.99 – -0.26 | **0.011** |  |  |  | -1.13 | -1.99 – -0.27 | **0.010** |
| Subjective Inequality |  |  |  |  |  |  | -0.12 | -0.56 – 0.32 | 0.586 | -0.52 | -1.00 – -0.03 | **0.037** | -0.02 | -0.47 – 0.42 | 0.912 | -0.52 | -1.00 – -0.03 | **0.037** |
| Unfairness Beliefs |  |  |  |  |  |  |  |  |  | 0.55 | 0.05 – 1.05 | **0.031** |  |  |  | 0.55 | 0.05 – 1.04 | **0.032** |
| Income * Subjective Inequality |  |  |  |  |  |  |  |  |  |  |  |  | -0.11 | -0.52 – 0.31 | 0.622 | -0.05 | -0.46 – 0.36 | 0.803 |
| Observations | 401 | | | 396 | | | 406 | | | 396 | | | 401 | | | 396 | | |
| R^2^ / R^2^ adjusted | 0.015 / 0.013 | | | 0.065 / 0.058 | | | 0.001 / -0.002 | | | 0.080 / 0.069 | | | 0.016 / 0.008 | | | 0.081 / 0.066 | | |

**Table S21.** Emotional intelligence predicted from income and subjective inequality including covariates in Study 2b. Conservatism: 1= very liberal, 7 = very conservative; Gender coded as 1 = male, 0 = female. Income: 1=$20,000 or less, 2=$20,00-$39,999, 3=$40,00-$59,999, 4=$60,00-$79,999, 5=$80,000-$99,999, 6=$100,000-$119,999, 7=$120,000-$159,999, 8=$160,000-$199,999, 9=$200,000-$299,999, 10=$300,000-$399,999, 11=$400,000 or above (measured as annual household income before tax). Income, conservatism, unfairness beliefs, and subjective inequality are standardized.

|  | **Emotional Intelligence Model 1** | | | **Emotional Intelligence Model 2** | | | **Emotional Intelligence Model 3** | | | **Emotional Intelligence Model 4** | | | **Emotional Intelligence Model 5** | | | **Emotional Intelligence Model 6** | | |
| --- | --- | --- | --- | --- | --- | --- | --- | --- | --- | --- | --- | --- | --- | --- | --- | --- | --- | --- |
| *Predictors* | *Estimates* | *CI* | *p* | *Estimates* | *CI* | *p* | *Estimates* | *CI* | *p* | *Estimates* | *CI* | *p* | *Estimates* | *CI* | *p* | *Estimates* | *CI* | *p* |
| (Intercept) | 25.75 | 25.31 – 26.20 | **<0.001** | 26.30 | 25.71 – 26.89 | **<0.001** | 25.73 | 25.30 – 26.17 | **<0.001** | 26.27 | 25.69 – 26.86 | **<0.001** | 25.75 | 25.30 – 26.20 | **<0.001** | 26.27 | 25.68 – 26.86 | **<0.001** |
| Years of Postsecondary Education | -0.03 | -0.48 – 0.41 | 0.886 | -0.15 | -0.58 – 0.29 | 0.514 |  |  |  | -0.14 | -0.57 – 0.30 | 0.538 | -0.02 | -0.47 – 0.42 | 0.922 | -0.13 | -0.57 – 0.30 | 0.545 |
| Conservatism |  |  |  | -0.83 | -1.27 – -0.39 | **<0.001** |  |  |  | -0.82 | -1.29 – -0.36 | **0.001** |  |  |  | -0.84 | -1.31 – -0.37 | **<0.001** |
| Gender |  |  |  | -1.16 | -2.04 – -0.29 | **0.009** |  |  |  | -1.10 | -1.98 – -0.23 | **0.013** |  |  |  | -1.11 | -1.99 – -0.24 | **0.012** |
| Subjective Inequality |  |  |  |  |  |  | -0.12 | -0.56 – 0.32 | 0.586 | -0.70 | -1.18 – -0.21 | **0.005** | -0.15 | -0.60 – 0.29 | 0.503 | -0.68 | -1.17 – -0.19 | **0.007** |
| Unfairness Beliefss |  |  |  |  |  |  |  |  |  | 0.60 | 0.10 – 1.11 | **0.019** |  |  |  | 0.60 | 0.09 – 1.10 | **0.021** |
| Years of Postsecondary Education * Subjective Inequality |  |  |  |  |  |  |  |  |  |  |  |  | 0.05 | -0.40 – 0.50 | 0.817 | 0.13 | -0.31 – 0.56 | 0.562 |
| Observations | 395 | | | 390 | | | 406 | | | 390 | | | 395 | | | 390 | | |
| R^2^ / R^2^ adjusted | 0.000 / -0.002 | | | 0.055 / 0.048 | | | 0.001 / -0.002 | | | 0.079 / 0.067 | | | 0.001 / -0.006 | | | 0.079 / 0.065 | | |

**Table S22.** Emotional intelligence predicted from years of postsecondary education and subjective inequality including covariates in Study 2b. Conservatism: 1= very liberal, 7 = very conservative; Gender coded as 1 = male, 0 = female. Years of postsecondary education: Participants were asked to indicate how many years of postsecondary education they have taken. Education, conservatism, unfairness beliefs, and subjective inequality are standardized.

|  | **Emotional Intelligence Model 1** | | | **Emotional Intelligence Model 2** | | | **Emotional Intelligence Model 3** | | | **Emotional Intelligence Model 4** | | | **Emotional Intelligence Model 5** | | | **Emotional Intelligence Model 6** | | |
| --- | --- | --- | --- | --- | --- | --- | --- | --- | --- | --- | --- | --- | --- | --- | --- | --- | --- | --- |
| *Predictors* | *Estimates* | *CI* | *p* | *Estimates* | *CI* | *p* | *Estimates* | *CI* | *p* | *Estimates* | *CI* | *p* | *Estimates* | *CI* | *p* | *Estimates* | *CI* | *p* |
| (Intercept) | 25.73 | 25.30 – 26.17 | **<0.001** | 26.29 | 25.71 – 26.87 | **<0.001** | 25.73 | 25.30 – 26.17 | **<0.001** | 26.26 | 25.68 – 26.84 | **<0.001** | 25.73 | 25.29 – 26.17 | **<0.001** | 26.26 | 25.68 – 26.84 | **<0.001** |
| Highest Degree | 0.13 | -0.31 – 0.57 | 0.554 | 0.07 | -0.35 – 0.50 | 0.737 |  |  |  | 0.06 | -0.37 – 0.48 | 0.795 | 0.14 | -0.30 – 0.57 | 0.541 | 0.06 | -0.37 – 0.48 | 0.786 |
| Conservatism |  |  |  | -0.77 | -1.21 – -0.34 | **<0.001** |  |  |  | -0.76 | -1.22 – -0.30 | **0.001** |  |  |  | -0.77 | -1.23 – -0.31 | **0.001** |
| Gender |  |  |  | -1.17 | -2.03 – -0.30 | **0.008** |  |  |  | -1.11 | -1.97 – -0.25 | **0.012** |  |  |  | -1.12 | -1.99 – -0.26 | **0.011** |
| Subjective Inequality |  |  |  |  |  |  | -0.12 | -0.56 – 0.32 | 0.586 | -0.61 | -1.09 – -0.13 | **0.012** | -0.11 | -0.55 – 0.33 | 0.618 | -0.60 | -1.08 – -0.11 | **0.016** |
| Unfairness Beliefs |  |  |  |  |  |  |  |  |  | 0.53 | 0.04 – 1.03 | **0.035** |  |  |  | 0.53 | 0.03 – 1.03 | **0.039** |
| Highest Degree * Subjective Inequality |  |  |  |  |  |  |  |  |  |  |  |  | 0.10 | -0.33 – 0.53 | 0.653 | 0.10 | -0.32 – 0.51 | 0.647 |
| Observations | 406 | | | 401 | | | 406 | | | 401 | | | 406 | | | 401 | | |
| R^2^ / R^2^ adjusted | 0.001 / -0.002 | | | 0.052 / 0.045 | | | 0.001 / -0.002 | | | 0.071 / 0.059 | | | 0.002 / -0.005 | | | 0.071 / 0.057 | | |

**Table S23.** Emotional intelligence predicted from highest degree and subjective inequality including covariates in Study 2b. Conservatism: 1= very liberal, 7 = very conservative; Gender coded as 1 = male, 0 = female. Degree: 1=no formal education, 2=elementary school, 3=junior high school, 4=senior high school or GED, 5=2-year college degree, 6=4-year college degree, 7=master's degree, 8=doctoral degree. Education, conservatism, unfairness beliefs, and subjective inequality are standardized.

|  | *Emotional*  *Intelligence* | *Inequality*  *Manipulation* | *Subjective*  *Socioeconomic*  *Status* | *Income* | *Degree* | *Years of*  *Postsecondary*  *Education* | *Subjective*  *Inequality* | *Unfairness*  *Beliefs* | *Conservatism* | *Age* | *Gender* |
| --- | --- | --- | --- | --- | --- | --- | --- | --- | --- | --- | --- |
| *Emotional*  *Intelligence* | .73 |  |  |  |  |  |  |  |  |  |  |
| *Inequality*  *Manipulation* | -0.008 *(.792)* | - |  |  |  |  |  |  |  |  |  |
| *Subjective*  *Socioeconomic*  *Status* | -0.129 *(<.001)* | -0.048 *(.137)* | - |  |  |  |  |  |  |  |  |
| *Income* | -0.056 *(.083)* | 0.007 *(.817)* | 0.464 *(<.001)* | - |  |  |  |  |  |  |  |
| *Degree* | 0.004 *(.896)* | -0.002 *(.955)* | 0.310 *(<.001)* | 0.245 *(<.001)* | - |  |  |  |  |  |  |
| *Years of*  *Postsecondary*  *Education* | -0.027 *(.399)* | -0.038 *(.236)* | 0.180 *(<.001)* | 0.106 *(.001)* | 0.584 *(<.001)* | - |  |  |  |  |  |
| *Subjective*  *Inequality* | 0.059 *(.069)* | 0.376 *(<.001)* | -0.188 *(<.001)* | -0.095 *(.003)* | 0.043 *(.178)* | 0.025 *(.445)* | - |  |  |  |  |
| *Unfairness*  *Beliefs* | 0.053 *(.099)* | 0.255 *(<.001)* | -0.258 *(<.001)* | -0.144 *(<.001)* | 0.024 *(.458)* | 0.029 *(.363)* | 0.756 *(<.001)* | - |  |  |  |
| *Conservatism* | -0.100 *(.002)* | -0.008 *(.809)* | 0.096 *(.003)* | 0.053 *(.098)* | -0.081 *(.012)* | -0.049 *(.125)* | -0.316 *(<.001)* | -0.329 *(<.001)* | - |  |  |
| *Age* | 0.122 *(<.001)* | -0.038 *(.239)* | 0.031 *(.334)* | 0.054 *(.091)* | 0.031 *(.341)* | 0.059 *(.067)* | 0.044 *(.170)* | -0.072 *(.025)* | 0.084 *(.009)* | - |  |
| *Gender* | -0.162 *(<.001)* | -0.060 *(.061)* | 0.034 *(.288)* | 0.031 *(.331)* | -0.030 *(.357)* | 0.018 *(.573)* | -0.086 *(.007)* | -0.109 *(.001)* | 0.040 *(.212)* | -0.079 *(.014)* | - |
| *Computed correlation used pearson-method with listwise-deletion.* | | | | | | | | | | | |

**Table S24.** Correlation between all measures (with Cronbach’s alpha on diagonal) in Study 3. Inequality Manipulation: 0 = low inequality, 1 = high inequality; Income: 1=$20,000 or less, 2=$20,00-$39,999, 3=$40,00-$59,999, 4=$60,00-$79,999, 5=$80,000-$99,999, 6=$100,000-$119,999, 7=$120,000-$159,999, 8=$160,000-$199,999, 9=$200,000-$299,999, 10=$300,000-$399,999, 11=$400,000 or above (measured as annual household income before tax); Degree: 1=no formal education, 2=elementary school, 3=junior high school, 4=senior high school or GED, 5=2-year college degree, 6=4-year college degree, 7=master's degree, 8=doctoral degree; Years of postsecondary education: Participants were asked to indicate how many years of postsecondary education they have taken. Conservatism: 1= very liberal, 7 = very conservative; Gender coded as 0 = female, 1 = male.

**
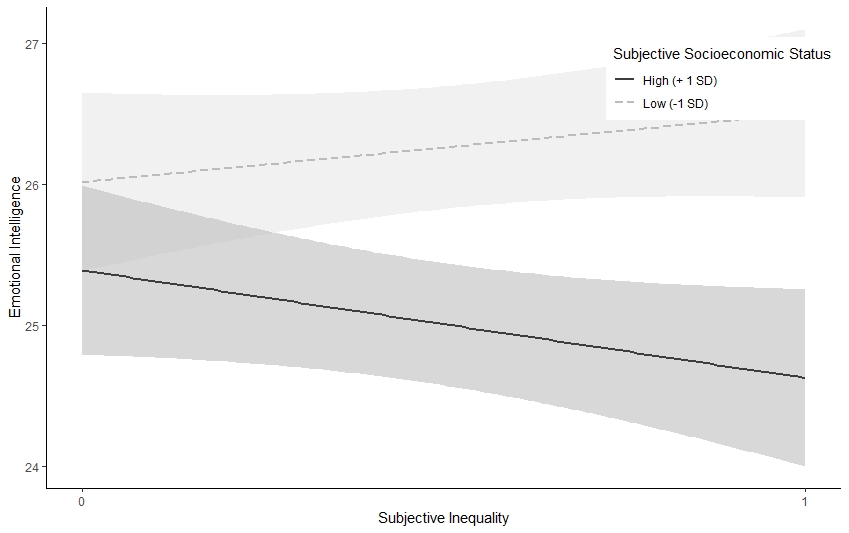
**

**Figure S4.** Association between inequality condition and emotional intelligence at + 1SD (b=-0.76, p.085) and at -1SD of SES (b=0.49, p=0.272) in Study 3. Intervals around regression lines are 95% confidence intervals. Condition: 0 = low inequality, 1 = high inequality.

|  | **Emotional Intelligence Model 1** | | | **Emotional Intelligence Model 2** | | | **Emotional Intelligence Model 3** | | | **Emotional Intelligence Model 4** | | | **Emotional Intelligence Model 5** | | | **Emotional Intelligence Model 6** | | |
| --- | --- | --- | --- | --- | --- | --- | --- | --- | --- | --- | --- | --- | --- | --- | --- | --- | --- | --- |
| *Predictors* | *Estimates* | *CI* | *p* | *Estimates* | *CI* | *p* | *Estimates* | *CI* | *p* | *Estimates* | *CI* | *p* | *Estimates* | *CI* | *p* | *Estimates* | *CI* | *p* |
| (Intercept) | 25.65 | 25.34 – 25.96 | **<0.001** | 26.23 | 25.85 – 26.61 | **<0.001** | 25.69 | 25.25 – 26.13 | **<0.001** | 26.35 | 25.85 – 26.85 | **<0.001** | 25.71 | 25.27 – 26.14 | **<0.001** | 26.32 | 25.83 – 26.82 | **<0.001** |
| Subjective SES | -0.62 | -0.93 – -0.32 | **<0.001** | -0.57 | -0.88 – -0.27 | **<0.001** |  |  |  | -0.58 | -0.88 – -0.27 | **<0.001** | -0.31 | -0.75 – 0.12 | 0.158 | -0.31 | -0.75 – 0.12 | 0.153 |
| Conservatism |  |  |  | -0.39 | -0.70 – -0.09 | **0.012** |  |  |  | -0.39 | -0.70 – -0.09 | **0.012** |  |  |  | -0.38 | -0.69 – -0.08 | **0.014** |
| Gender |  |  |  | -1.56 | -2.19 – -0.93 | **<0.001** |  |  |  | -1.58 | -2.21 – -0.95 | **<0.001** |  |  |  | -1.55 | -2.18 – -0.92 | **<0.001** |
| Condition |  |  |  |  |  |  | -0.08 | -0.70 – 0.54 | 0.806 | -0.22 | -0.83 – 0.38 | 0.472 | -0.14 | -0.75 – 0.47 | 0.658 | -0.22 | -0.83 – 0.39 | 0.478 |
| Subjective SES*Condition |  |  |  |  |  |  |  |  |  |  |  |  | -0.62 | -1.24 – -0.01 | **0.046** | -0.52 | -1.13 – 0.09 | 0.094 |
| Observations | 972 | | | 965 | | | 972 | | | 965 | | | 972 | | | 965 | | |
| R^2^ / adjusted R^2^ | 0.016 / 0.015 | | | 0.047 / 0.044 | | | 0.000 / -0.001 | | | 0.048 / 0.044 | | | 0.020 / 0.017 | | | 0.051 / 0.046 | | |

**Table S25.** Emotional intelligence predicted from SES and inequality manipulation including covariates in Study 3. Conservatism: 1= very liberal, 7 = very conservative; Gender coded as 1 = male, 0 = female; SES = subjective socioeconomic status; Condition: 0= low inequality, 1 = high inequality. Conservatism and SES are standardized.

|  | **Emotional Intelligence Model 1** | | | **Emotional Intelligence Model 2** | | | **Emotional Intelligence Model 3** | | | **Emotional Intelligence Model 4** | | | **Emotional Intelligence Model 5** | | | **Emotional Intelligence Model 6** | | |
| --- | --- | --- | --- | --- | --- | --- | --- | --- | --- | --- | --- | --- | --- | --- | --- | --- | --- | --- |
| *Predictors* | *Estimates* | *CI* | *p* | *Estimates* | *CI* | *p* | *Estimates* | *CI* | *p* | *Estimates* | *CI* | *p* | *Estimates* | *CI* | *p* | *Estimates* | *CI* | *p* |
| (Intercept) | 26.16 | 25.84 – 26.48 | **<0.001** | 26.73 | 26.34 – 27.13 | **<0.001** | 26.55 | 26.05 – 27.04 | **<0.001** | 27.23 | 26.68 – 27.78 | **<0.001** | 26.53 | 26.03 – 27.02 | **<0.001** | 27.16 | 26.60 – 27.71 | **<0.001** |
| Subjective SES | -0.29 | -0.62 – 0.04 | 0.083 | -0.24 | -0.57 – 0.09 | 0.155 |  |  |  | -0.28 | -0.61 – 0.05 | 0.091 | 0.16 | -0.36 – 0.68 | 0.554 | 0.14 | -0.38 – 0.66 | 0.600 |
| Conservatism |  |  |  | -0.30 | -0.62 – 0.03 | 0.075 |  |  |  | -0.31 | -0.63 – 0.02 | 0.064 |  |  |  | -0.29 | -0.62 – 0.03 | 0.075 |
| Gender |  |  |  | -1.63 | -2.29 – -0.96 | **<0.001** |  |  |  | -1.67 | -2.33 – -1.01 | **<0.001** |  |  |  | -1.63 | -2.29 – -0.97 | **<0.001** |
| Condition |  |  |  |  |  |  | -0.66 | -1.31 – -0.02 | **0.045** | -0.83 | -1.47 – -0.18 | **0.012** | -0.71 | -1.36 – -0.06 | **0.033** | -0.80 | -1.44 – -0.15 | **0.015** |
| Subjective SES*Condition |  |  |  |  |  |  |  |  |  |  |  |  | -0.82 | -1.49 – -0.14 | **0.018** | -0.70 | -1.37 – -0.03 | **0.041** |
| Observations | 771 | | | 764 | | | 771 | | | 764 | | | 771 | | | 764 | | |
| R^2^ / adjusted R^2^ | 0.004 / 0.003 | | | 0.038 / 0.034 | | | 0.005 / 0.004 | | | 0.046 / 0.041 | | | 0.018 / 0.014 | | | 0.051 / 0.045 | | |

**Table S26.** Additional exploratory analysis: Emotional intelligence predicted from SES and inequality manipulation after applying additional exclusion criteria in Study 3. SES = socioeconomic status; Condition coded as 0 = low inequality, 1 = high inequality; Gender coded as 1 = male, 0 = female. Conservatism, and SES are standardized. Participants were asked to describe in 1-3 sentences how the society they live in is relatively low (low inequality condition) or high (high inequality condition). Two research assistants who were blind to the hypothesis and conditions, coded the responses as either describing inequality as low, high, or unclear. Participants who failed to provide a response in line with the video they watched or who stated that they disagreed with the arguments made in the video were excluded.

|  | **Emotional Intelligence Model 1** | | | **Emotional Intelligence Model 2** | | | **Emotional Intelligence Model 3** | | | **Emotional Intelligence Model 4** | | | **Emotional Intelligence Model 5** | | | **Emotional Intelligence Model 6** | | |
| --- | --- | --- | --- | --- | --- | --- | --- | --- | --- | --- | --- | --- | --- | --- | --- | --- | --- | --- |
| *Predictors* | *Estimates* | *CI* | *p* | *Estimates* | *CI* | *p* | *Estimates* | *CI* | *p* | *Estimates* | *CI* | *p* | *Estimates* | *CI* | *p* | *Estimates* | *CI* | *p* |
| (Intercept) | 26.16 | 25.84 – 26.48 | **<0.001** | 26.38 | 25.98 – 26.78 | **<0.001** | 25.71 | 25.25 – 26.17 | **<0.001** | 26.40 | 25.88 – 26.93 | **<0.001** | 25.74 | 25.27 – 26.20 | **<0.001** | 26.38 | 25.85 – 26.90 | **<0.001** |
| Subjective SES | -0.29 | -0.62 – 0.04 | 0.083 | -0.44 | -0.76 – -0.12 | **0.008** |  |  |  | -0.44 | -0.77 – -0.12 | **0.008** | -0.24 | -0.71 – 0.22 | 0.306 | -0.24 | -0.70 – 0.23 | 0.319 |
| Conservatism |  |  |  | -0.43 | -0.76 – -0.10 | **0.011** |  |  |  | -0.43 | -0.76 – -0.10 | **0.011** |  |  |  | -0.43 | -0.76 – -0.10 | **0.012** |
| Gender |  |  |  | -1.59 | -2.25 – -0.92 | **<0.001** |  |  |  | -1.59 | -2.26 – -0.92 | **<0.001** |  |  |  | -1.58 | -2.25 – -0.91 | **<0.001** |
| Condition |  |  |  |  |  |  | 0.21 | -0.44 – 0.86 | 0.523 | -0.05 | -0.70 – 0.59 | 0.875 | 0.11 | -0.54 – 0.76 | 0.742 | -0.04 | -0.69 – 0.60 | 0.892 |
| Subjective SES*Condition |  |  |  |  |  |  |  |  |  |  |  |  | -0.48 | -1.14 – 0.17 | 0.150 | -0.41 | -1.05 – 0.24 | 0.222 |
| Observations | 771 | | | 830 | | | 836 | | | 830 | | | 836 | | | 830 | | |
| R^2^ / adjusted R^2^ | 0.004 / 0.003 | | | 0.046 / 0.042 | | | 0.000 / -0.001 | | | 0.046 / 0.041 | | | 0.013 / 0.010 | | | 0.047 / 0.042 | | |

**Table S27.** Additional exploratory analysis: Emotional intelligence predicted from SES and inequality manipulation after applying additional exclusion criteria in Study 3. SES = socioeconomic status; Condition coded as 0 = low inequality, 1 = high inequality; Gender coded as 1 = male, 0 = female. Conservatism, and SES are standardized. Participants whose response to the manipulation check questions was much higher (low inequality condition) or much lower (high inequality condition) than that of the majority of participants in their own condition were excluded. We excluded participants whose response was more than 1SD above or below (in the two conditions, respectively) that of their group mean.

|  | **Emotional Intelligence Model 1** | | | **Emotional Intelligence Model 2** | | | **Emotional Intelligence Model 3** | | | **Emotional Intelligence Model 4** | | | **Emotional Intelligence Model 5** | | | **Emotional Intelligence Model 6** | | |
| --- | --- | --- | --- | --- | --- | --- | --- | --- | --- | --- | --- | --- | --- | --- | --- | --- | --- | --- |
| *Predictors* | *Estimates* | *CI* | *p* | *Estimates* | *CI* | *p* | *Estimates* | *CI* | *p* | *Estimates* | *CI* | *p* | *Estimates* | *CI* | *p* | *Estimates* | *CI* | *p* |
| (Intercept) | 25.65 | 25.34 – 25.96 | **<0.001** | 26.24 | 25.85 – 26.62 | **<0.001** | 25.69 | 25.25 – 26.13 | **<0.001** | 26.33 | 25.82 – 26.83 | **<0.001** | 25.69 | 25.25 – 26.13 | **<0.001** | 26.33 | 25.82 – 26.83 | **<0.001** |
| Income | -0.31 | -0.62 – 0.00 | 0.051 | -0.22 | -0.52 – 0.09 | 0.170 |  |  |  | -0.22 | -0.52 – 0.09 | 0.171 | -0.17 | -0.61 – 0.27 | 0.456 | -0.06 | -0.51 – 0.38 | 0.774 |
| Conservatism |  |  |  | -0.43 | -0.74 – -0.13 | **0.005** |  |  |  | -0.43 | -0.74 – -0.13 | **0.005** |  |  |  | -0.43 | -0.74 – -0.13 | **0.006** |
| Gender |  |  |  | -1.59 | -2.22 – -0.95 | **<0.001** |  |  |  | -1.60 | -2.23 – -0.96 | **<0.001** |  |  |  | -1.59 | -2.23 – -0.96 | **<0.001** |
| Condition |  |  |  |  |  |  | -0.08 | -0.70 – 0.54 | 0.806 | -0.17 | -0.78 – 0.44 | 0.589 | -0.07 | -0.69 – 0.54 | 0.814 | -0.17 | -0.78 – 0.44 | 0.587 |
| Income*Condition |  |  |  |  |  |  |  |  |  |  |  |  | -0.27 | -0.89 – 0.34 | 0.385 | -0.29 | -0.91 – 0.32 | 0.352 |
| Observations | 972 | | | 965 | | | 972 | | | 965 | | | 972 | | | 965 | | |
| R^2^ / R^2^ adjusted | 0.004 / 0.003 | | | 0.036 / 0.033 | | | 0.000 / -0.001 | | | 0.036 / 0.032 | | | 0.005 / 0.002 | | | 0.037 / 0.032 | | |

**Table S28.** Emotional intelligence predicted from income and inequality manipulation including covariates in Study 3. Conservatism: 1= very liberal, 7 = very conservative; Gender coded as 1 = male, 0 = Condition: 0= low inequality, 1 = high inequality. Income: 1=$20,000 or less, 2=$20,00-$39,999, 3=$40,00-$59,999, 4=$60,00-$79,999, 5=$80,000-$99,999, 6=$100,000-$119,999, 7=$120,000-$159,999, 8=$160,000-$199,999, 9=$200,000-$299,999, 10=$300,000-$399,999, 11=$400,000 or above (measured as annual household income before tax). Conservatism and income are standardized.

|  | **Emotional Intelligence Model 1** | | | **Emotional Intelligence Model 2** | | | **Emotional Intelligence Model 3** | | | **Emotional Intelligence Model 4** | | | **Emotional Intelligence Model 5** | | | **Emotional Intelligence Model 6** | | |
| --- | --- | --- | --- | --- | --- | --- | --- | --- | --- | --- | --- | --- | --- | --- | --- | --- | --- | --- |
| *Predictors* | *Estimates* | *CI* | *p* | *Estimates* | *CI* | *p* | *Estimates* | *CI* | *p* | *Estimates* | *CI* | *p* | *Estimates* | *CI* | *p* | *Estimates* | *CI* | *p* |
| (Intercept) | 25.65 | 25.34 – 25.96 | **<0.001** | 26.25 | 25.86 – 26.63 | **<0.001** | 25.69 | 25.25 – 26.13 | **<0.001** | 26.34 | 25.84 – 26.84 | **<0.001** | 25.69 | 25.25 – 26.13 | **<0.001** | 26.33 | 25.83 – 26.83 | **<0.001** |
| Years of Postsecondary Education | -0.15 | -0.46 – 0.16 | 0.341 | -0.14 | -0.44 – 0.17 | 0.381 |  |  |  | -0.14 | -0.45 – 0.17 | 0.370 | 0.01 | -0.45 – 0.46 | 0.977 | -0.03 | -0.48 – 0.42 | 0.896 |
| Conservatism |  |  |  | -0.45 | -0.76 – -0.14 | **0.004** |  |  |  | -0.45 | -0.76 – -0.15 | **0.004** |  |  |  | -0.45 | -0.76 – -0.15 | **0.004** |
| Gender |  |  |  | -1.60 | -2.23 – -0.97 | **<0.001** |  |  |  | -1.61 | -2.24 – -0.98 | **<0.001** |  |  |  | -1.60 | -2.24 – -0.96 | **<0.001** |
| Condition |  |  |  |  |  |  | -0.08 | -0.70 – 0.54 | 0.806 | -0.18 | -0.79 – 0.44 | 0.575 | -0.08 | -0.70 – 0.54 | 0.793 | -0.17 | -0.79 – 0.44 | 0.578 |
| Years of Postsecondary Education *Condition |  |  |  |  |  |  |  |  |  |  |  |  | -0.29 | -0.92 – 0.33 | 0.355 | -0.21 | -0.82 – 0.41 | 0.513 |
| Observations | 970 | | | 963 | | | 972 | | | 963 | | | 970 | | | 963 | | |
| R^2^ / R^2^ adjusted | 0.001 / -0.000 | | | 0.035 / 0.032 | | | 0.000 / -0.001 | | | 0.035 / 0.031 | | | 0.002 / -0.001 | | | 0.036 / 0.031 | | |

**Table S29.** Emotional intelligence predicted from years of postsecondary education and inequality manipulation including covariates in Study 3. Conservatism: 1= very liberal, 7 = very conservative; Gender coded as 1 = male, 0 = Condition: 0= low inequality, 1 = high inequality. Years of postsecondary education: Participants were asked to indicate how many years of postsecondary education they have taken. Conservatism and education are standardized.

|  | **Emotional Intelligence Model 1** | | | **Emotional Intelligence Model 2** | | | **Emotional Intelligence Model 3** | | | **Emotional Intelligence Model 4** | | | **Emotional Intelligence Model 5** | | | **Emotional Intelligence Model 6** | | |
| --- | --- | --- | --- | --- | --- | --- | --- | --- | --- | --- | --- | --- | --- | --- | --- | --- | --- | --- |
| *Predictors* | *Estimates* | *CI* | *p* | *Estimates* | *CI* | *p* | *Estimates* | *CI* | *p* | *Estimates* | *CI* | *p* | *Estimates* | *CI* | *p* | *Estimates* | *CI* | *p* |
| (Intercept) | 25.65 | 25.34 – 25.96 | **<0.001** | 26.24 | 25.86 – 26.63 | **<0.001** | 25.69 | 25.25 – 26.13 | **<0.001** | 26.33 | 25.83 – 26.84 | **<0.001** | 25.69 | 25.25 – 26.13 | **<0.001** | 26.34 | 25.83 – 26.84 | **<0.001** |
| Highest Degree | 0.01 | -0.30 – 0.32 | 0.955 | -0.03 | -0.34 – 0.28 | 0.835 |  |  |  | -0.03 | -0.34 – 0.27 | 0.834 | -0.05 | -0.50 – 0.39 | 0.814 | -0.11 | -0.55 – 0.33 | 0.621 |
| Conservatism |  |  |  | -0.45 | -0.75 – -0.14 | **0.004** |  |  |  | -0.45 | -0.76 – -0.14 | **0.004** |  |  |  | -0.44 | -0.75 – -0.14 | **0.005** |
| Gender |  |  |  | -1.60 | -2.23 – -0.97 | **<0.001** |  |  |  | -1.61 | -2.25 – -0.98 | **<0.001** |  |  |  | -1.62 | -2.25 – -0.98 | **<0.001** |
| Condition |  |  |  |  |  |  | -0.08 | -0.70 – 0.54 | 0.806 | -0.17 | -0.78 – 0.44 | 0.582 | -0.08 | -0.70 – 0.54 | 0.806 | -0.17 | -0.79 – 0.44 | 0.581 |
| Highest Degree *Condition |  |  |  |  |  |  |  |  |  |  |  |  | 0.12 | -0.50 – 0.74 | 0.704 | 0.15 | -0.46 – 0.77 | 0.627 |
| Observations | 972 | | | 965 | | | 972 | | | 965 | | | 972 | | | 965 | | |
| R^2^ / R^2^ adjusted | 0.000 / -0.001 | | | 0.034 / 0.031 | | | 0.000 / -0.001 | | | 0.034 / 0.030 | | | 0.000 / -0.003 | | | 0.035 / 0.030 | | |

**Table S30.** Emotional intelligence predicted from highest degree and inequality manipulation including covariates in Study 3. Conservatism: 1= very liberal, 7 = very conservative; Gender coded as 1 = male, 0 = Condition: 0= low inequality, 1 = high inequality.); Degree: 1=no formal education, 2=elementary school, 3=junior high school, 4=senior high school or GED, 5=2-year college degree, 6=4-year college degree, 7=master's degree, 8=doctoral degree. Conservatism and education are standardized.

**
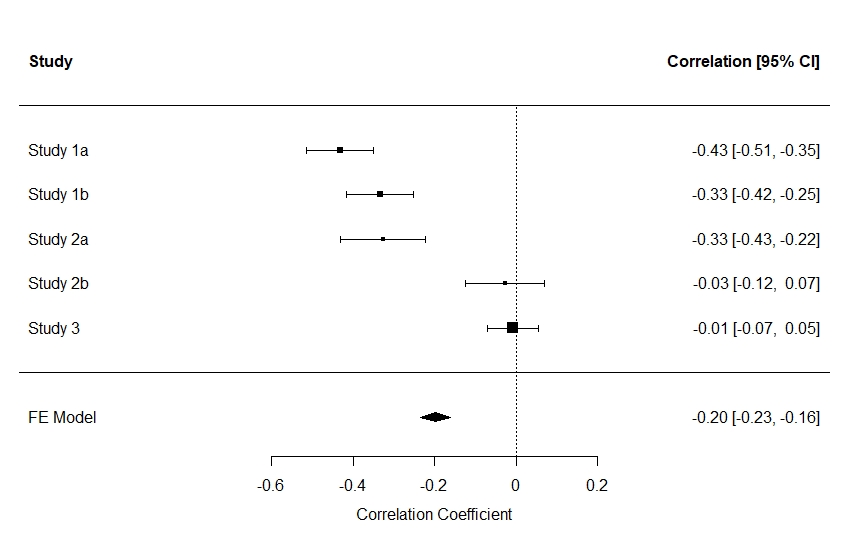
**

**Figure S5.** Internal meta-analysis of the relationship between subjective economic inequality and emotional intelligence. Forest plot showing the correlation between subjective economic inequality and emotional intelligence. The size of each square in the forest plot is proportional to the weight of that sample. The estimate for the fixed-effects model is also given. CI=confidence interval.

**Procedure for all meta-analyses.** We used the estimates for each study that we obtained from the models as described in the manuscript and converted them into correlations for the main effect of subjective inequality and into partial correlations for the interaction using the metafor package (Viechtbauer, 2010). All meta-analyses were conducted both without and with covariates. We note that we haven’t conducted other studies on this question with these methods.

**
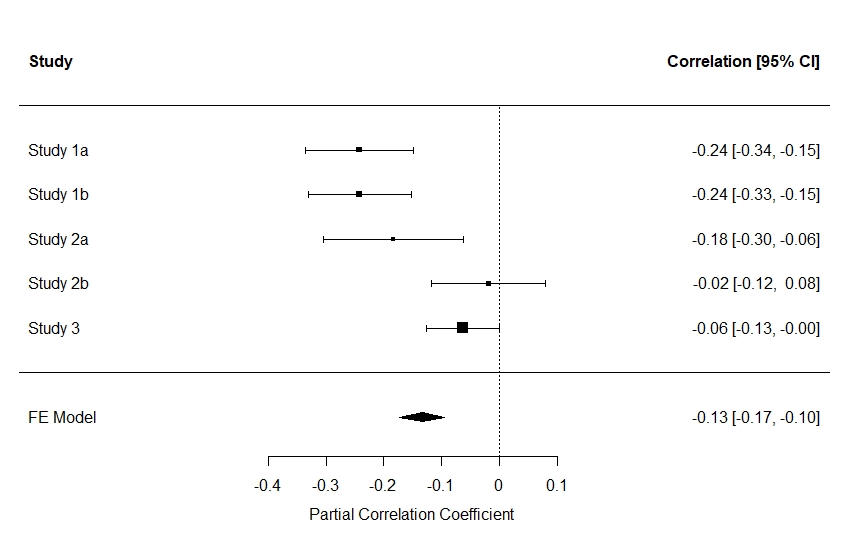
Figure S6.** Internal meta-analysis of the interaction between SES and subjective economic inequality in predicting emotional intelligence. Forest plot showing the partial correlation between the interaction of SES and subjective economic inequality in predicting emotional intelligence. The size of each square in the forest plot is proportional to the weight of that sample. The estimate for the fixed-effects model is also given. CI=confidence interval.

**
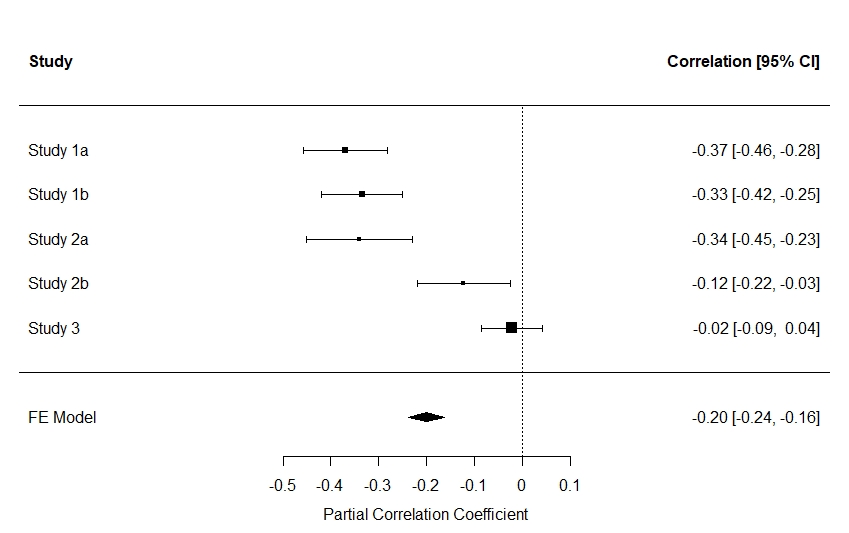
**

**Figure S7.** Internal meta-analysis of the relationship between subjective economic inequality and emotional intelligence. Forest plot showing the partial correlation between subjective economic inequality and emotional intelligence controlling for conservatism, gender, SES, and unfairness beliefs (the latter in Studies 1a, 1b, 2a, and 2b only), *r_partial_* = -.20, *z* =-10.46, *p* < .001, 95% CI [-0.24, -0.16]. The size of each square in the forest plot is proportional to the weight of that sample. The estimate for the fixed-effects model is also given. CI = confidence interval.


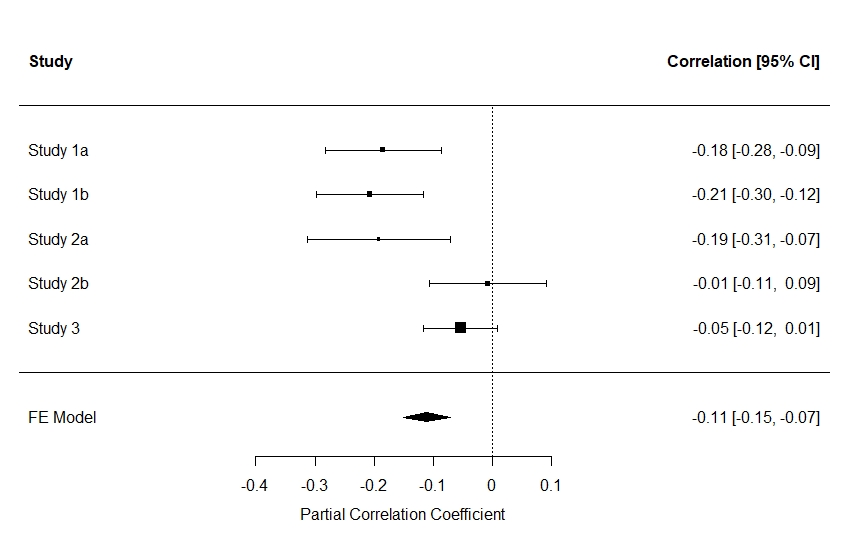


**Figure S8.** Internal meta-analysis of the interaction between SES and subjective economic inequality in predicting emotional intelligence. Forest plot showing the partial correlation between the interaction of SES and subjective economic inequality in predicting emotional intelligence controlling for conservatism, gender, SES, and unfairness beliefs (the latter in Studies 1a, 1b, 2a, and 2b only), *r_partial_* = -.11, *z* =-5.53, *p* < .001, 95% CI [-0.15, -0.07]. The size of each square in the forest plot is proportional to the weight of that sample. The estimate for the fixed-effects model is also given. CI = confidence interval.


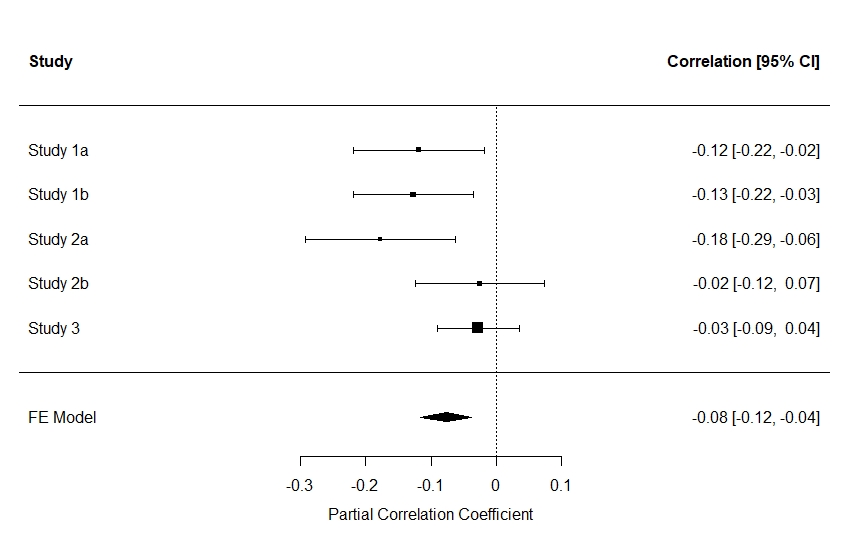


**Figure S9.** Internal meta-analysis of the interaction between income and subjective economic inequality in predicting emotional intelligence. Forest plot showing the partial correlation between the interaction of SES and subjective economic inequality in predicting emotional intelligence, *r_partial_* = -.08, *z* =-3.84, *p* < .001, 95% CI [-0.12, -0.04]. The size of each square in the forest plot is proportional to the weight of that sample. The estimate for the fixed-effects model is also given. CI = confidence interval.

**
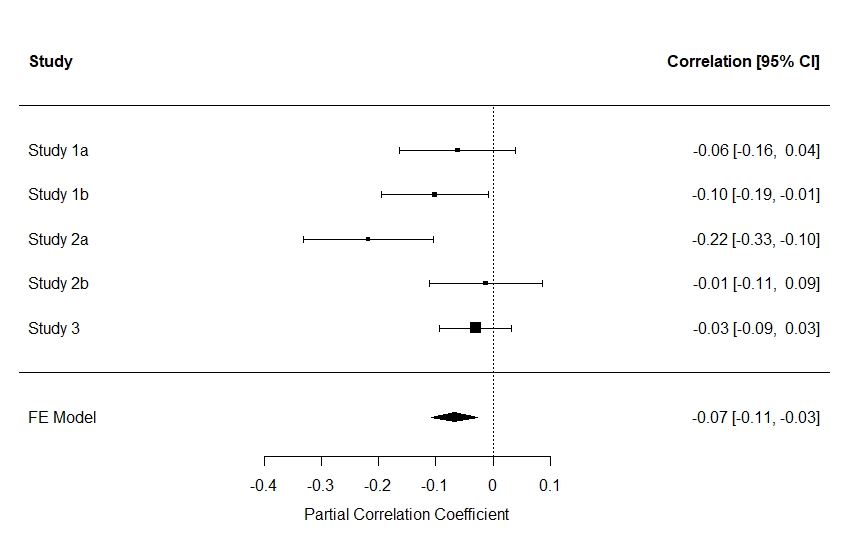
**

**Figure S10.** Internal meta-analysis of the interaction between income and subjective economic inequality in predicting emotional intelligence. Forest plot showing the partial correlation between the interaction of SES and subjective economic inequality in predicting emotional intelligence controlling for conservatism, gender, SES, and unfairness beliefs (the latter in Studies 1a, 1b, 2a, and 2b only), *r_partial_* = -.07, *z* =-3.35, *p* < .001, 95% CI [-0.11, -0.03]. The size of each square in the forest plot is proportional to the weight of that sample. The estimate for the fixed-effects model is also given. CI = confidence interval.


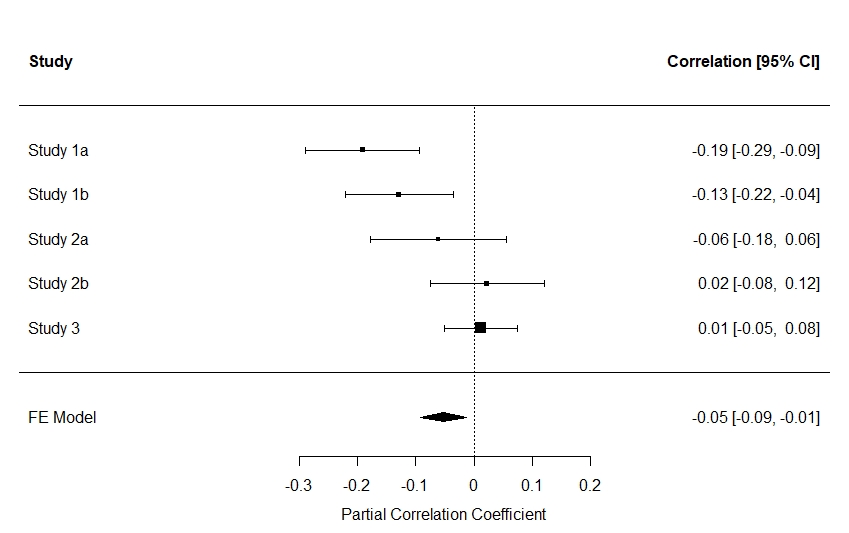


**Figure S11.** Internal meta-analysis of the interaction between degree and subjective economic inequality in predicting emotional intelligence. Forest plot showing the partial correlation between the interaction of SES and subjective economic inequality in predicting emotional intelligence, *r_partial_* = -.05, *z* =-2.62, *p* = .009, 95% CI [-0.09, -0.01]. The size of each square in the forest plot is proportional to the weight of that sample. The estimate for the fixed-effects model is also given. CI = confidence interval.

**
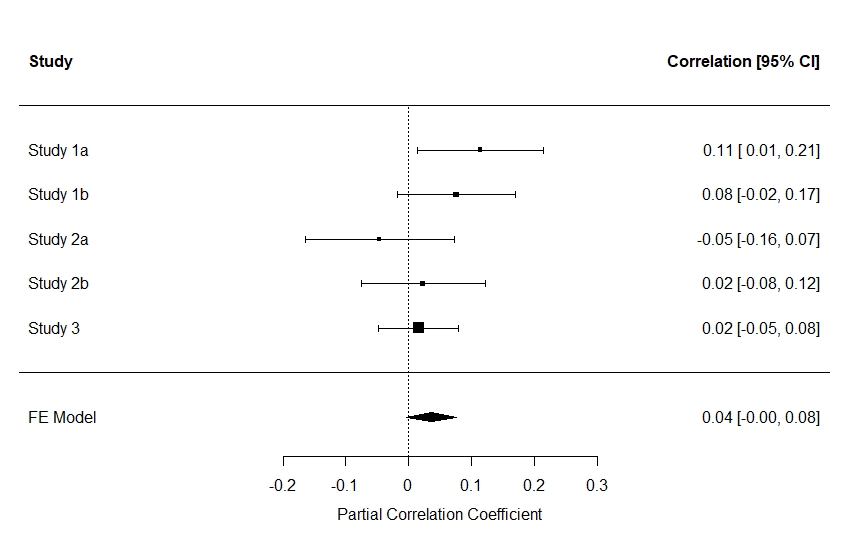
**

**Figure S12.** Internal meta-analysis of the interaction between degree and subjective economic inequality in predicting emotional intelligence. Forest plot showing the partial correlation between the interaction of SES and subjective economic inequality in predicting emotional intelligence controlling for conservatism, gender, SES, and unfairness beliefs (the latter in Studies 1a, 1b, 2a, and 2b only), *r_partial_* = .04, *z* =1.78, *p* = .075, 95% CI [-0.004, 0.08]. The size of each square in the forest plot is proportional to the weight of that sample. The estimate for the fixed-effects model is also given. CI = confidence interval.

**
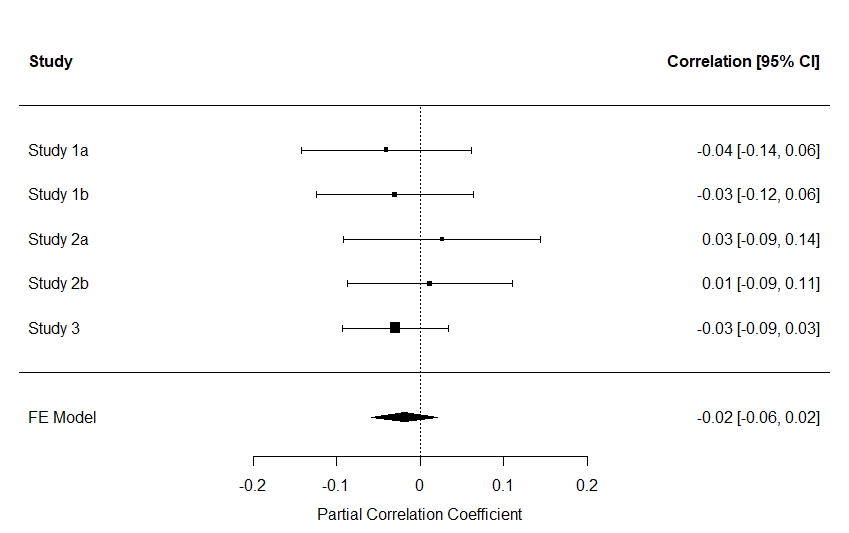
**

**Figure S13.** Internal meta-analysis of the interaction between years of postsecondary education and subjective economic inequality in predicting emotional intelligence. Forest plot showing the partial correlation between the interaction of SES and subjective economic inequality in predicting emotional intelligence, *r_partial_* = -.02, *z* =-0.92, *p* = .357, 95% CI [-0.06, 0.02]. The size of each square in the forest plot is proportional to the weight of that sample. The estimate for the fixed-effects model is also given. CI = confidence interval.

**
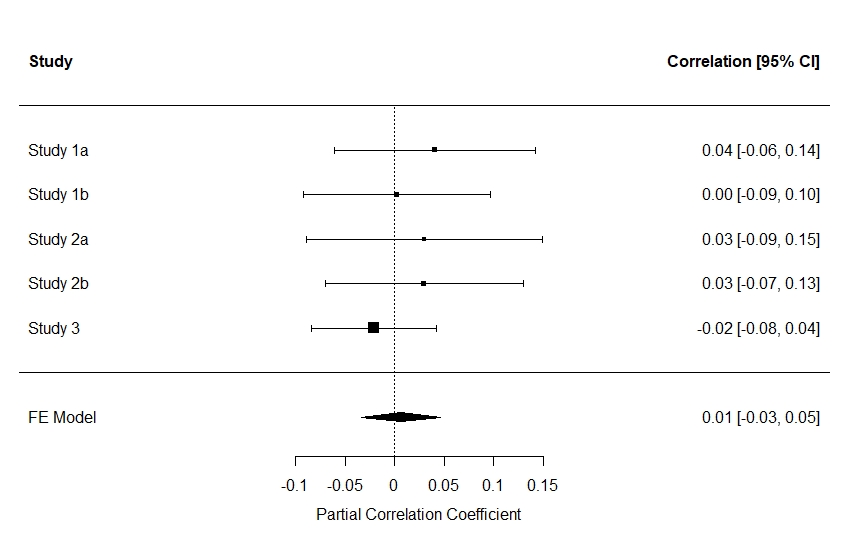
**

**Figure S14.** Internal meta-analysis of the interaction between years of postsecondary education and subjective economic inequality in predicting emotional intelligence. Forest plot showing the partial correlation between the interaction of SES and subjective economic inequality in predicting emotional intelligence controlling for conservatism, gender, SES, and unfairness beliefs (the latter in Studies 1a, 1b, 2a, and 2b only), *r_partial_* = .01, *z* =0.31, *p* = .758, 95% CI [-0.03, 0.05]. The size of each square in the forest plot is proportional to the weight of that sample. The estimate for the fixed-effects model is also given. CI = confidence interval.

References

Giner-Sorolla, R., Carpenter, T., Lewis, N. A., Montoya, A. K., Aberson, C. L., Bostyn, D. H., et al. (2021). Power to detect what? Considerations for planning and evaluating sample size. Unpublished manuscript.

Hughes, J. (2017). paramtest: Run a function iteratively while varying

parameters. R package version. 0.1.0. https://CRAN.R-project.org/package=paramtest

Viechtbauer, W. (2010). Conducting meta-analyses in R with the metafor

package. Journal of Statistical Software, 36(3), 1–48.
